# Performance of diabetes risk prediction models: a systematic review and meta-analysis

**DOI:** 10.1530/EC-25-0353

**Published:** 2025-11-03

**Authors:** Meichen Li, Aowei Tan, Linjie Song, Xingying Qiu, Li Zhou, Zehuai Wen

**Affiliations:** ^1^ Second Clinical Medical College of Guangzhou University of Chinese Medicine, Guangzhou, China; ^2^ State Key Laboratory of Dampness Syndrome of Chinese Medicine, Second Affiliated Hospital of Guangzhou University of Chinese Medicine, Guangzhou, China; ^3^ Center for Clinical Research, Second Affiliated Hospital of Guangzhou University of Chinese Medicine, Guangzhou, China

**Keywords:** type 2 diabetes mellitus, risk prediction models, meta-analysis, machine learning, screening

## Abstract

**Background:**

Type 2 diabetes mellitus (T2DM) poses a significant global public health burden, where early detection of at-risk populations is imperative for implementing targeted preventive strategies. This systematic review and meta-analysis aimed to evaluate the methodological quality and predictive performance of existing T2DM risk prediction models in screening contexts.

**Methods:**

Following the TRIPOD-SRMA statement, eligible studies were selected through searching seven databases (CNKI, WanFang Database, VIP, PubMed, Embase, Web of Science, and the Cochrane Library) from database inception through December 2024. Methodological quality was assessed using the PROBAST tool. Random-effects models synthesized discrimination (AUC). Subgroup analyses explored geographic, modeling, and validation-related heterogeneity. Funnel plots and Egger’s regression test assessed small-study effects.

**Results:**

A total of 65 studies (encompassing 97 distinct prediction models) were included in the analysis. Among 97 models, logistic regression dominated (97.9% of models), achieving moderate discrimination (AUC: 0.628–0.916), while machine learning (ML) models showed marginally higher AUCs (up to 0.998). Geographic and cohort disparities emerged, with USA-based models outperforming others (USA AUC: 0.97 vs China AUC: 0.79) and poor performance in prediabetic cohorts (AUC: 0.72 vs 0.80 in normoglycemic). External validation remained limited (21 models), though spatial/temporal validation cohorts demonstrated stable performance. High risk of bias and application (>80% of models) stemmed from inadequate statistical reporting and external verification definitions.

**Conclusion:**

ML has favorable diagnostic accuracy for the progression of T2DM. This provides evidence for the development of predictive tools with broader applicability. Future research should prioritize external validation to enhance precision.

## Introduction

The global burden of type 2 diabetes mellitus (T2DM) has reached epidemic proportions, with an estimated 537 million adults affected worldwide in 2021, a figure projected to rise to 783 million by 2045 (1). First-line pharmacological interventions demonstrate limited long-term efficacy due to progressive β-cell dysfunction and acquired drug resistance (2). This therapeutic challenge underscores the critical importance of preventive strategies and early detection in mitigating the devastating microvascular and macrovascular complications associated with undiagnosed hyperglycemia (3). Advances in predictive medicine have generated multiple risk stratification tools integrating variables spanning anthropometric indices and biochemical markers (4). However, the selection of optimal diabetes prediction models poses significant challenges due to methodological heterogeneity among existing tools, such as analytical frameworks (machine learning (ML) algorithms versus conventional regression analyses), predictor selection criteria (clinically accessible parameters versus specialized biomarkers), and validation protocols (internal versus external cohort verification) (5, 6). The inherent methodological heterogeneity introduces critical trade-offs among model complexity, generalizability, and clinical feasibility, with each computational approach presenting distinct strengths and limitations (7). Therefore, there is a need to refine systematic reviews of diabetes prediction models to facilitate the selection of the optimal predictive model.

Existing systematic reviews of T2DM prediction models require updating to address fundamental limitations in existing evidence. While conventional regression-based methodologies have been extensively examined in prior analyses (8), contemporary ML developments, particularly deep neural networks and ensemble techniques increasingly employed in clinical investigations, lack comprehensive evaluation (9). Moreover, emerging evidence underscores significant disparities in model performance across ethnic populations, healthcare settings, novel predictors, and social determinants of health (10, 11, 12). However, current meta-analytic approaches remain insufficient in analyzing this multidimensional heterogeneity spanning geographical regions and demographic subgroups. The prediction model risk of bias assessment tool (PROBAST) framework enables a more comprehensive methodological appraisal of prediction models to identify superior modeling approaches (5), which have also not been comprehensively appraised in existing reviews. These gaps compromise the translational relevance of current evidence, necessitating an updated synthesis aligned with the PRISMA statement (13).

Therefore, this study conducts a systematic review to identify, critically evaluate, and synthesize the methodological quality and predictive performance of existing T2DM prediction models, aiming to provide evidence-based recommendations for optimizing the development and clinical application of future risk stratification tools.

## Methods

This study adhered to the Transparent Reporting of a Multivariable Prediction Model for Individual Prognosis or Diagnosis – Systematic Review and Meta-analysis (TRIPOD-SRMA) statement (14). The review protocol was registered on the International Prospective Register of Systematic Reviews (PROSPERO) with registration ID CRD42024569731.

### Study selection

The following eligibility criteria were used for considering studies for this review. The inclusion criteria were as follows: i) population: adults aged ≥18 years with T2DM; ii) outcomes: development or validation of T2DM risk prediction models; and iii) study design: cohort studies (prospective or retrospective). The exclusion criteria were i) studies investigating T2DM risk factors without model development, ii) non-Chinese/English publications, and iii) cross-sectional studies, case–control studies, or studies with incomplete data/full-text unavailability.

### Search strategy

Electronic searches were conducted in the following databases from inception to December 2024: i) Chinese literature databases: CNKI, WanFang Database, and VIP Database; and ii) English databases: PubMed, Embase, Web of Science, and the Cochrane Library. Search terms combined medical subject headings (MeSH) and free-text keywords adapted to database-specific syntax. Example English terms included: diabete*; type 2 diabetes; T2DM; prediction model; prognostic model; risk prediction; risk score; and risk assessment.

The following PubMed search terms were an example of the search strategy:

#1: search (‘type 2 diabetes’[mesh]) OR (‘diabetes mellitus type II’ or ‘diabetes mellitus type 2’ or ‘type 2 diabetes’ or ‘type 2 diabetes mellitus’ or ‘diabetes type 2’ or ‘T2DM’ or ‘diabete*’).

#2: search (‘predictive model’[mesh]) OR (‘prediction model’ or ‘clinical risk score’ or ‘clinical prediction model’ or ‘clinical predictive model’ or ‘clinical scoring system’ or ‘risk assessment model’ or ‘risk prediction model’ or ‘risk predictive model’).

#3: #1 AND #2.

### Study screening and data extraction

Study screening and data extraction were independently performed by two investigators (Meichen Li and Aowei Tan) with cross-verification according to the eligibility criteria. Discrepancies were resolved through consensus discussions or adjudication by a third reviewer (Li Zhou). Data extraction followed the Critical Appraisal and Data Extraction for Systematic Reviews of Prediction Modelling Studies (CHARMS) checklist (15), with results tabulated to capture: i) study characteristics, including first author, country, and study design (development/validation); ii) methodological parameters: sample size, handling of missing data, model-building techniques (e.g., regression algorithms), and variable selection methods (stepwise/LASSO); and iii) model performance metrics: discrimination (area under the curve statistics), calibration (observed/expected ratios), and presentation format (nomograms/risk scores).

### Risk of bias and applicability assessment

Two investigators (Meichen Li and Aowei Tan) independently evaluated the methodological quality of included studies using the PROBAST tool (4), assessing four domains: i) participant selection: evaluates whether the study population appropriately represents the target clinical population, including inclusion/exclusion criteria clarity and potential selection bias (e.g., inappropriate exclusions or incomplete enrollment). ii) Predictor variables: examines the definition, measurement, and timing of predictors, including whether predictors were clearly defined and measured before outcome assessment (blinding of predictor assessment). iii) Outcome definition: assesses the clinical relevance and objectivity of the outcome, including the handling of missing data (e.g., exclusion, imputation, or transparency in reporting). iv) Statistical analysis: critically appraises model development and validation methods, covering the handling of overfitting, model calibration/discrimination metrics, and internal/external validation procedures. Each domain received three-tiered risk classifications: low risk, unclear risk, and high risk.

In addition, overall applicability (clinical relevance) was graded across the first three domains to evaluate whether the study’s design and execution aligned with real-world clinical practice: i) participant selection: whether the study population matched the intended clinical setting. ii) Predictor variables: feasibility of obtaining predictors in routine care. (iii) Outcome definition: clinical utility of the outcome measure. Each domain was classified as low, unclear, or high concern for bias risk and real-world implementability. Discrepancies between reviewers were resolved through iterative discussion until consensus was reached, with a third senior researcher (Zhou Li) arbitrating unresolved cases.

### Data synthesis and analysis

The methodological framework for evaluating predictive model performance was established in accordance with the TRIPOD-SRMA statement for systematic reviews and meta-analyses of prediction models (14). Data synthesis was performed by pooling discrimination metrics (AUC-ROC values) across studies using Hartung-Knapp-Sidik-Jonkman (HKSJ) random-effects models, with between-study variance estimated via restricted maximum likelihood (REML) (16). For the primary metric, in addition to reporting point estimates, 95% confidence intervals (CI) were reported. Heterogeneity assessment was conducted using Cochran’s *Q*-test and quantified with the *I*^2^ statistic, where *I*^2^ > 50% indicated substantial heterogeneity. Predefined subgroup analyses were performed to explore heterogeneity sources, including country (geographic origin of study populations), model type (e.g., logistic regression (LR) vs ML), number of predictors (<10 vs ≥ 10), study timeframe (data collection period), and cohort size stratification (<1,000 vs ≥ 1,000 participants). Sensitivity analyses included leave-one-out cross-validation to evaluate the robustness of pooled estimates and iterative exclusion of studies. Reporting bias was assessed using funnel plots complemented by Egger’s regression test for small-study effects when ≥10 studies were included in a meta-analysis. Sensitivity analysis was performed by sequentially omitting each study to assess the robustness of the pooled effect size. For clinical interpretability, decision curve analysis (DCA) was implemented for externally validated models to evaluate net benefit across threshold probabilities. Net benefit was calculated as the proportion of true positives minus the proportion of false positives weighted by the odds of the selected risk threshold. Moreover, co-occurrence network analysis visualized predictors using force-directed algorithms to identify clusters of covariate associations. All statistical analyses were conducted in R v4.1.0 (metafor, dmetar, rmda, and igraph packages).

## Results

### Study identification

**Figure 1:**
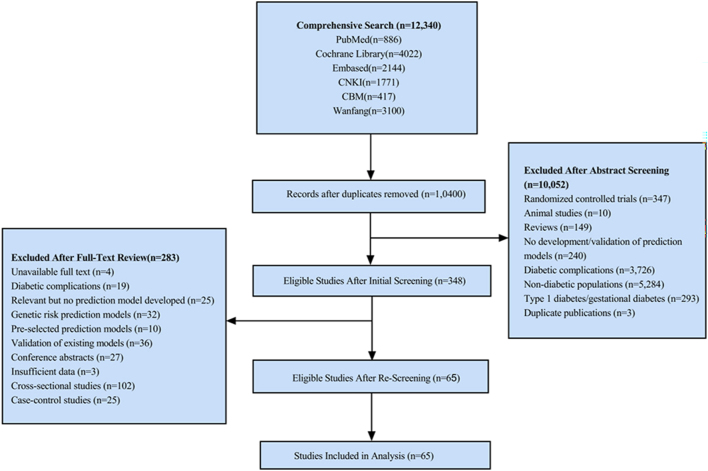
Search results and study selection. TC: total cholesterol; FPG: fasting plasma glucose; TG: triglycerides; HDL: high-density lipoprotein; BMI: body mass index; WC: waist circumference; BP: blood pressure; WHR: waist-to-hip ratio; CVD: cardiovascular disease; PDM: pre-diabetes mellitus; OGTT: oral glucose tolerance test; Glu: glucose; HbA1c: glycated hemoglobin; SCR: serum creatinine; ALT: alanine transaminase; LDL: low-density lipoprotein; SBP: systolic blood pressure; DBP: diastolic blood pressure.

The preliminary search identified a total of 12,340 publications across six databases: CNKI (*n* = 1,771), WanFang (*n* = 3,100), CBM (*n* = 417), PubMed (*n* = 887), Embase (*n* = 2,144), and the Cochrane Library (*n* = 4,021). Following database searching, records underwent two-stage screening. Title/abstract screening excluded 10,052 records. The remaining records underwent full-text assessment. Full-text review excluded 283 records. Following a systematic screening process, 65 studies (encompassing 97 distinct prediction models) were identified for inclusion in the analysis. The literature screening flowchart and detailed results are depicted in Fig. 1.

### Study characteristics

The 65 included studies (97 models) encompassed diverse geographic regions, predominantly China (47.4%), followed by Japan (13.4%), South Korea (9.3%), the USA (7.2%), India (6.2%), Iran (2.06%), England (2.06%), and European countries (2.06%). Study populations comprised non-diabetic individuals (91.8%) and prediabetic participants (8.2%), with follow-up durations ranging from 1 to 20 years, most commonly 5 years (35.1%). Age groups varied widely, including adults aged ≥18 years to elderly populations (≥60 years). Sample sizes spanned from 487 to 2,540,753 participants, with outcome events (e.g., diabetes incidence) reported across cohorts. Of the 97 models, 15 (15.5%) were derived from retrospective cohort studies, and 82 (84.5%) from prospective cohort studies. The characteristics of the included studies are presented in Table 1.

**Table 1:** Characteristics of model development studies.

Study	Country	Study population	Follow-up	Age	Sample size	Outcome events	Predictor variables content	Missing data
Han 2017 (23)	China	Non-DM	5-year	≥50	17,690	1,309	BMI; WC; FPG; HBP; lipid disorders; smoke; family history of DM	Deletion
Sun 2013 (1) (21)	China	Not-DM	5-year	35–70	T: 12,392	T: 361	Age; family history of DM; height; WC; FPG; TG; HDL-C; TC	–
					EV: 4,215	EV: 154		
Sun 2013 (2) (21)	China	Not-DM	5-year	35–70	T: 22,936	T: 220	Age; family history of DM; height; WC; FPG; TG; HDL-C; TC	–
					IV: 3,229	IV: 99		
Sun 2013 (3) (21)	China	Not-DM	5-year	35–70	T: 12,392	T: 361	Age; family history of DM; height; WC; FPG; TG; HDL-C; TC	–
					EV: 3,344	EV: 139		
Cai 2020 (24)	China	Not-DM	5-year	≥20	T: 22,936	T: 220	FPG; TC; TG; HDL-C; LDL-C; smoke; alcohol; family history of DM	No missing data
					IV: 9,830	IV: 190		
Hippisley-Cox 2009 (1) (18)	England & Wales	Not-DM	10-year	25–79	Male	Male	Race; age; sex; BMI; smoke; family history of DM; tmvnsend; HBP; CVD; medication history	Multiple imputation
					T: 2,540,753	T: 78,081		
					EV: 1,232,832	EV: 3,7535		
Hippisley-Cox 2009 (2) (18)	England & Wales	Not-DM	10-year	25–79	Female	Female	Race; age; sex; BMI; smoke; family history of DM; tmvnsend; HBP; CVD; medication history	Multiple imputation
					T: 2,540,753	T: 78,081		
					EV: 1,232,832	EV: 37,535		
Wen 2016 (1) (25)	China	Not-DM	6-year	≥30	T: 2,754	T: 144	Age; BMI; WC; family history of DM	Expectation-maximization
					IV: 1,378	IV: 74		
Wen 2016 (2) (25)	China	Not-DM	6-year	≥30	T: 2,754	T: 144	Age; BMI; WC; family history of DM; FPG; TG	Expectation-maximization
					IV: 1,378	IV: 74		
Wang 2018 (26)	China	Not-DM	6-year	≥18	5,706	194	Age; diet; BMI; FPG; TG	No missing data
Bai 2018 (27)	China	Not-DM	5-year	18–90	T: 8,238	T: 1,197	Sex; age; BMI; smoke; alcohol; HBP; family history of HBP/DM; SBP; DBP; FPG; TC; TG; fatty liver	–
					IV: 3,531	IV: 496		
Lei 2023 (28)	China	Not-DM	5-year	≥60	T: 17,363	T: 1,072	Age; FPG; BMI; SBP; TG; ALT	Deletion
					IV: 7,441	IV: 468		
Fu 2023 (29)	China	Not-DM	7-year	≥60	712	33	Sex; age; BMI; ALT; TG; HbA1c; FPG	Deletion
Su 2017 (1) (30)	China	Not-DM	3-year	20–70	Male	Male	Age; BMI; FPG; TG; ALT; WBC	–
					18,963	1,044		
Su 2017 (2) (30)	China	Not-DM	3-year	20–70	Female	Female	Age; FPG; TG; HDL-C; ALT	–
					14,482	580		
Zhang 2022 (31)	China	Not-DM	3-year	>60	1,512	173	Age; BMI; WC; BP; smoke; alcohol; family history of DM; Glu; insulin; HDL-C; TG	–
Yang 2016 (32)	China	Not-DM	5-year	20–80	16,715	858	Age; BMI; FPG; TG; HBP; WBC	–
Zhang 2016 (1) (33)	China	Not-DM	6-year	≥18	Non-invasive	Non-invasive	Sex; age; education; smoke; sleep; BMI; WC; HBP; TC; TG; HDL-C; FPG	–
					T: 6,143	T: 376		
					IV: 6,142	IV: 403		
Zhang 2016 (2) (33)	China	Not-DM	6-year	≥18	Invasive	Invasive	HBP; TC; TG; HDL-C; FPG	–
					T: 6,143	T: 376		
					IV: 6,142	IV: 403		
Zhu 2023 (1) (34)	China	Not-DM	3-year	≥18	T: 1,133	425	Age; sex; education; smoke; alcohol; medication history; SBP; DBP; TC; LDL; HDL-C; TG; ALT; UA; WC; WHR; BMI; lipid disorders,ect	Deletion
					IV: 284			
Zhu 2023 (2) (34)	China	PDM	3-year	≥18	T: 796	360	Age; sex; education; medication history; smoke; alcohol; SBP; DBP; TC; LDL; HDL-C; TG; ALT; UA; WC; WHR; BMI; lipid disorders,ect	Deletion
					IV: 199			
Chen 2018 (35)	China	PDM	1-year	≥18	T: 3,803	T: 99	Age; BMI; HBP; diet	–
					IV: 1,853	IV: 57		
Ma 2020 (36)	China	Not-DM	2-year	≥30	3,127	187	Smoke; medication history; OGTT; FPG; BMI; family history of DM	Deletion
Liu 2020 (37)	China	Not-DM	6-year	≥18	7,289	562	Age; sex; family history of DM; BMI; SBP; LDL-C; FPG	Multiple imputation
Lee 2024 (1) (38)	South Korea	Not-DM	8-year	40–69	T: 5,459	T: 460	Age; BMI,; HTN; HDL-C; TG; FPG; HbA1c; γ-GTP	Multiple imputation
					IV: 2,318	IV: 180		
					EV: 2,043	EV: 368		
					EV: 4,091	EV: 138		
Lee 2024 (2) (38)	South Korea	Not-DM	8-year	40–69	T: 5,459	T: 460	Age; BMI,; HTN; HDL-C; TG; FPG; HbA1c; γ-GTP	Multiple imputation
					IV: 2,318	IV: 180		
					EV: 2,043	EV: 368		
					EV: 4,091	EV: 138		
Alssema 2011 (39)	Europe	Not-DM	5-year	≥45	18,301	844	Age; BMI; WC; medication history; sex; history of GMD; smoke; family history of DM	Multiple imputation
Li 2019 (40)	China	Not-DM	5-year	≥40	17,173	1,159	Age; sex,; FPG; BMI,; WC; TC; TG; BP; family history of DM; ALT	Deletion
Gao 2009 (1) (41)	India	Not-DM	10-year	20–65	Male	Male	Obesity; BMI; WC; BP; TG; family history of DM; FPG	Deletion
					T: 1,141	T: 259		
					IV: 1,182	IV: 259		
Gao 2009 (2) (41)	India	Not-DM	10-year	20–65	Female	Female	Obesity; BMI; WC; BP; TG; family history of DM; FPG	Deletion
					T: 1,953	T: 252		
					IV: 1,550	IV: 245		
Edlitz 2022 (1) (20)	USA	Not-DM	5-year	40–69	IV: 7,948	IV: 64	Visit interval; height; HBP; exercise; smoke; diet; family history of DM	Iterative imputation method
					EV: 10,064	EV: 201		
Edlitz 2022 (2) (20)	USA	PDM	5-year	40–69	IV: 1,006	IV: 94	Visit interval; height; HBP; exercise; smoke; diet; family history of DM	Iterative imputation method
					EV: 7,059	EV: 501		
Katsimpris 2021 (42)	Germany	Not-DM	5-year	40–69	1,591	139	Age; sex; BMI; diet	–
Liu 2016 (43)	China	Not-DM	20-year	≥55	1,857	144	Sex; age; BMI; FPG; self-assessment of health; exercise; disability; marital status; lipid disorders; education; diet	Deletion
Hafezi 2024 (44)	Iran	Not-DM	10-year	≥55	T: 5,033	T: 942	LAP; BMI; VAI	–
					IV: 1,677	IV: 314		
Hu 2020 (45)	China	Not-DM	5-year	40–69	4,833	171	Age; sex; smoke; exercise; BMI; TG,FPG	–
Xu 2014 (46)	China	Not-DM	4-year	≥50	T: 8,000	1,063	Family history of DM; BMI; BP; TC; FPG; HDL	Inverse probability weighting
					IV: 8,043			
Rathmann 2010 (47)	Germany	Not-DM	7-year	55–74	887	93	Age; sex; BMI; family history of DM; smoke; HBP; FPG; HbA1c; UA; OGTT	–
Liu 2022 (48)	China	Not-DM	2-year	≥65	T: 10,1625	8,298	Age; gender; education; marital status; BP; exercise; smoke; alcohol; WC; SBP; FPG; TC; TG; HDL-C; LDL-C; ALT; AST; TBIL; Scr; BUN; SUA	–
					IV: 25,406			
Zhang 2020 (49)	Australia	Not-DM	10-year	≥45	T: 165,609	T: 10,019	Age; sex; BMI; education; family history of DM; residence; income; BP; CVD; lipid disorders; smoke; exercise; diet	–
					IV: 70,975	IV: 4,294		
Alghamdi 2017 (50)	USA	Not-DM	5-year	≥18	32,555	5,099	Age; HR; metabolic equivalent; SBP; DBP; exercise; race; BMI; lipid disorders; medication history; CVD	–
Wang 2018 (1) (51)	China	Not-DM	3-year	≥18	Male	Male	Metabolic syndrome; BMI; IGR; FPG; OGTT; BP; lipid disorders; TG; HDL-C	Deletion
					5,087	801		
Wang 2018 (2) (51)	China	Not-DM	3-year	≥18	Female	Female	Metabolic syndrome; BMI; IGR; FPG; OGTT; BP; lipid disorders; TG; HDL-C	Deletion
					3,549	298		
Sun 2023 (52)	China	Not-DM	7-year	35–60	2,755	172	Age; BMl; WC; lipid disorders; HBP; FPG; family history of DM; RvD1; RvD2	–
Stern 2002 (53)	USA	Not-DM	7-year	25–64	2,903	269	Age; sex; race; FPG; SBP; HDL-CTC; family history of DM; BMI; OGTT	–
Tran Quang 2022 (54)	Vietnam	Not-DM	5-year	40–64	1,448	140	Region; sleep; SBP; WC; FPG	–
Sun 2016 (55)	China	Not-DM	6-year	50–70	2,103	507	Age; sex; region; smoke; alcohol; exercise; family history of DM; BMI; FPG; HbA1c; SBP	–
Heianza 2012 (1) (56)	Japan	Not-DM	5-year	40–75	T: 7,654	T: 289	NLA (Age; sex; family history of DM; smoke; BMI)	Deletion
					EV: 1,976	EV: 57		
Heianza 2012 (2) (56)	Japan	Not-DM	5-year	40–75	T: 7,654	T: 289	NLA; FPG; HbA1c	Deletion
					EV: 1,976	EV: 57		
Heianza 2012 (3) (56)	Japan	Not-DM	5-year	40–75	T: 7,654	T: 289	NLA; FPG	Deletion
					EV: 1,976	EV: 57		
Heianza 2012 (4) (56)	Japan	Not-DM	5-year	40–75	T: 7,654	T: 289	NLA; HbA1c	Deletion
					EV: 1,976	EV: 57		
Oh 2021 (1) (57)	South Korea	Not-DM	10-year	40–69	T: 3,973	T: 910	KDR (age; region; smoke; family history of DM; WC)	–
					IV: 1,700	IV: 381		
Oh 2021 (2) (57)	South Korea	Not-DM	10-year	40–69	T: 3,973	T: 910	KDR; FPG	–
					IV: 1,700	IV: 381		
Oh 2021 (3) (57)	South Korea	Not-DM	10-year	40–69	T: 3,973	T: 910	KDR; HbA1c	–
					IV: 1,700	IV: 381		
Nicolaisen 2022 (58)	Denmark	PDM	5-year	≥30	T: 20,806	T: 2,449	HbA1c; age; sex; BMI; BP; diet; self-assessment of physical health	Deletion
					IV: 5,201	IV: –		
Hu 2018 (1) (59)	Japan	Not-DM	7-year	30–59	T: 30,500	T: 2216	Age; sex; abdominal obesity; BMI; smoke; BP; lipid disorders; HbA1C; FPG	Deletion
					IV: 13,349	IV: 1,169		
Hu 2018 (2) (59)	Japan	Not-DM	7-year	30–59	T: 30,500	T: 2,216	Age; sex; abdominal obesity; BMI; smoke; HBP	Deletion
					IV: 13,349	IV: 1,169		
Xu 2024 (1) (60)	Japan	Not-DM	5-year	46–75	T: 10,986	T: 707	Sex; BMI; family history of DM; DBP	Multiple imputation
					EV: 11,345	EV: 673		
Xu 2024 (2) (60)	Japan	Not-DM	5-year	46–75	T: 10,986	T: 707	HbA1c; family history of DM	Multiple imputation
					EV: 11,345	EV: 673		
Xu 2024 (3) (60)	Japan	Not-DM	5-year	46–75	T: 10,986	T: 707	FPG; HbA1c; family history of DM	Multiple imputation
					EV: 11,345	EV: 673		
Arellano-Campos 2019 (61)	Mexico	Not-DM	3-year	≥20	6,144	331	Age; T; IFG; HBP; abdominal obesity	–
Shao 2020 (1) (62)	China	Not-DM	10-year	≥20	T: 4,498	T: 257	Age; sex; race; HBP; smoke; alcohol; WC; BMI	Deletion
					IV: 1,525	IV: 92		
Shao 2020 (2) (62)	China	Not-DM	10-year	≥20	T: 4,498	T: 257	Age; sex; race; BP; smoke; alcohol; WC; BMI; education; exercise; diet; triceps skinfold thickness; sleep	Deletion
					IV: 1,525	IV: 92		
Shao 2020 (3) (62)	China	Not-DM	10-year	≥20	T: 4,498	T: 257	Age; sex; race; HBP; smoke; alcohol; WC; BMI; education; exercise; diet; triceps skinfold thickness; sleep; LDL; HDL; TC; TG; insulin; FPG; HbA1c	Deletion
					IV: 1,525	IV: 92		
Shao 2020 (4) (62)	China	Not-DM	10-year	≥20	T: 4,498	T: 257	LDL; HDL; TC; TG; insulin; FPG; HbA1c	Deletion
					IV: 1,525	IV: 92		
Rhee 2021 (63)	South Korea	Not-DM	10-year	≥40	T: 268,241	T: 23,420	FPG; age; sex; ALT; BMI; GGT; SBP; TC; AST; alcohol	Multiple imputation
					IV: 67,061	IV: 5,736		
Cai 2021 (64)	Japan	Not-DM	5-year	Mean 43.64	T: 9,651	T: 154	Age; fatty liver; GGT; TG; HbA1c; FPG	–
					IV: 3,289	IV: 47		
Olivera 2017 (65)	Brazil	Not-DM	10-year	35–74	T: 3,709	1,359	Exercise; alcohol; education; age; BMI; medication history; WHR; income; CVD; diet; family history of HBP; family history of DM; sex	Deletion
					IV: 8,738			
Liu 2024 (66)	USA	Not-DM	5-year	≥35	T: 32,372	T: 411	Age; sex; race; BMI; SBP; HDL-C; TC; family history of DM	Deletion
					IV: 13,875	IV: 205		
Jagannathan 2020 (1) (67)	India	PDM	3-year	Mean 44.6	548	167	Basic model (FPG; age; sex; family history of DM; BMI; SBP; TG; HDL-C)	Deletion
Jagannathan 2020 (2) (67)	India	PDM	3-year	Mean 44.6	548	167	Basic model + IGT	Deletion
Jagannathan 2020 (3) (67)	India	PDM	3-year	Mean 44.6	548	167	Basic model; 30 min -PG > 182 mg/dl	Deletion
Jagannathan 2020 (4) (67)	India	PDM	3-year	Mean 44.6	548	167	Basic model; FPG; 30 min-PG > 182 mg/dl; I11T	Deletion
Savolainen 2017 (68)	Sweden	Not-DM	5-year	64	629	69	Non-invasive (WC; alcohol; smoke; SBP; family history of DM)	–
Sun 2009 (69)	Taiwan, China	Not-DM	5-year	35–74	T: 10,294	T: 483	Sex; education; age; smoke; BMI; WC	Deletion
					IV: 10,257	IV: 441		
Sun 2009 (69)	Taiwan, China	Not-DM	5-year	35–74	T: 10,294	T: 483	Sex; education; age; smoke; BMI; WC; FPG	Deletion
					IV: 10,257	IV: 441		
Lim 2012 (1) (70)	South Korea	Not-DM	4-year	40–69	6,342	436	Age; family history of DM; smoke; BMI; HBP	Deletion
Lim 2012 (2) (70)	South Korea	Not-DM	4-year	40–69	6,342	436	FPG; HDL-C; TG; age; family history of DM; smoke; BMI; HBP	Deletion
Lim 2012 (3) (70)	South Korea	Not-DM	4-year	40–69	6,342	436	HbA1c; FPG; HDL-C; TG; age; family history of DM; smoke; BMI; HBP	Deletion
Wang 2020 (71)	Japan	Not-DM	5-year	20–75	T: 8,296	T: 275	Age; WC; smoke; fatty liver; FPG; HbA1c	Deletion
					IV: 2,817	IV: 98		
Tan 2022 (72)	Japan	Not-DM	6-year	30–60	T: 1,518	T: 10	WC; HbA1c; FPG; HDL-C; TG; smoke	Deletion
					IV: 494	IV: 4		
Yatsuya 2018 (73)	Japan	Not-DM	10-year	35–64	2,960	548	Age; FPG; BMI; TG; WBC; HDL-C; TC	Deletion
Wang 2022 (1) (74)	China	Not-DM	4-year	Mean 61	15,934	1,302	BP; IFG; WC	Deletion
Wang 2022 (2) (74)	China	Not-DM	4-year	Mean 61	Male	Male	BP; IFG; WC	Deletion
					4,324	341		
Wang 2022 (3) (74)	China	Not-DM	4-year	Mean 61	Female	Female	BP; IFG; WC	Deletion
					11,610	961		
Wang 2022 (4) (74)	China	Not-DM	4-year	Mean 61	15,934	1,302	Sex; age; family history of DM; BMI; HBP; HDL-C; TG; FPG; WC	Deletion
Wilson 2007 (75)	USA	Not-DM	8-year	Mean 54	3,140	160	Age, sex, family history of DM, WC, BMI, BP, HDL-C, FPG, HOMA-IR	–
Yan 2020 (19)	China	Not-DM	5-year	55 ± 13	T: 810	T: 110	Age; SBP; WC; family history of DM	Deletion
					EV: 792	EV: 93		
Liu 2012 (1) (76)	China	Not-DM	5-year	25–75	Female	Female	Age; BMI; WC; SBP; FPG; family history of DM; smoke	–
					596	70		
Liu 2012 (2) (76)	China	Not-DM	5-year	25–75	Male	Male	Age; BMI; WC; SBP; FPG; family history of DM; smoke	–
					487	51		
Woo 2016 (77)	Hong Kong, China	Not-DM	9-year	Mean 50.3	1,380	123	Age; family history of DM; smoke; HBP; BMI; FPG	–
Jiang 2023 (78)	China	Not-DM	7-year	50–75	25,2176	63,423	BMI; age; SBP; DBP; diet; exercise	Deletion
Asgari 2021 (79)	Iran	Not-DM	9-year	≥20	T: 5,291	T: 214	Age; WC; height; SBP; TG; HDL-C; FPG; FHDM	Multiple imputation
					EV: 3,147	EV: 54		
Stiglic 2021 (80)	Europe	Not-DM	10-year	≥50	16,363	6,192	Age; SEX; BMI; alcohol; exercise	–
Lindström 2003 (81)	Finland	Not-DM	10-year	35–64	T: 4,746	T: 196	Age; BMI; WC; medication history; exercise; Glu; diet	Deletion
					EV: 4,615	EV: 67		
Schmidt 2005 (82)	USA	Not-DM	9-year	45–64	T: 3,958	T: 646	WC; height; HBP; SBP; DBP; family history of DM; race; age; BMI; FPG; HDL-C; TC; TG	Deletion
					IV: 3,957	IV: 646		
Chen 2010 (83)	Australia	Not-DM	5-year	>25	T: 6,060	T: 362	Age; sex; race; family history of DM; Glu; medication history; smoke; exercise; C	Multiple imputation
					EV: 2,393	EV: 115		
					EV: 6,034	EV: 320		

T, training; IV, internal validation; EV, external validation; DM, diabetes mellitus; BMI, body mass index; WC, waist circumference; FPG, fasting plasma glucose; HBP, high blood pressure; DBP, diastolic blood pressure; SBP, systolic blood pressure; TG, triglycerides; HDL-C, high-density lipoprotein cholesterol; TC, total cholesterol; LDL-C, low-density lipoprotein cholesterol; ALT, alanine aminotransferase; AST, aspartate aminotransferase; BUN, blood urea nitrogen; Scr, serum creatinine; SUA, serum uric acid; OGTT, oral glucose tolerance test; HbA1c, hemoglobin A1c; WBC, white blood cell count; HOMA-IR, homeostatic model assessment of insulin resistance; QUICKI, quantitative insulin sensitivity check index; GGT, gamma-glutamyl transferase; UA, uric acid; WHR, waist-to-hip ratio; LAP, lipid accumulation product; VAI, visceral adiposity index; CVD, cardiovascular disease; IGR, impaired glucose regulation; IFG, impaired fasting glucose; RvD1/RvD2, resolvin D1/D2; γ-GTP, gamma-glutamyl transpeptidase.

### Predictive model development

The included studies predominantly employed LR as the primary modeling method (97.9% of models). A subset of models (11.3%) utilized advanced ML techniques, such as random forest (RF) and XGBoost, predominantly in larger cohorts or when modeling complex variable interactions. The top ten predictors ranked by incorporation frequency were as follows: noninvasive predictors: age (69.1%), body mass index (BMI) (64.9%), family history of diabetes (44.3%), waist circumference (WC) (39.2%), smoking status (36.1%), diastolic blood pressure (DBP) (36.1%), sex (34.0%), systolic blood pressure (SBP) (21.6%), exercise (16.5%), diet (14.4), and alcohol consumption (14.4%); noninvasive predictors/invasive predictors (e.g., laboratory biomarkers): age (69.1%), BMI (64.9%), fasting plasma glucose (FPG) (58.8%), family history of diabetes (44.3%), WC (39.2%), triglycerides (TG) (38.1%), smoking status (36.1%), DBP (36.1%), sex (34.0%), and high-density lipoprotein (HDL) (29.9%).

Missing data handling methods, though inconsistently reported, included deletion (e.g., complete-case analysis), multiple imputation, or inverse probability weighting. However, many studies (34.02%) omitted explicit descriptions of data preprocessing. Model development frequently involved partitioning datasets into training and validation subsets (e.g., random split, bootstrap), while a minority applied cross-validation (e.g., five-fold, ten-fold) to optimize generalizability. The predictive model development of the included studies is presented in Table 1.

### Network relationships of diabetes predictors

**Figure 2:**
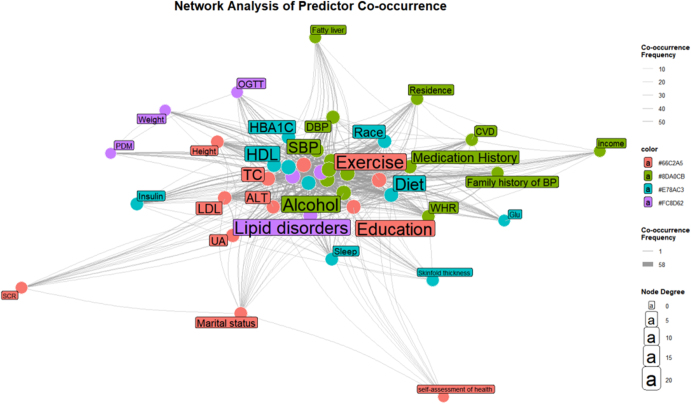
Predictor co-occurrence network. Node size reflects the frequency of each predictor in the cohort; line thickness indicates how often two predictors co-occur within the same participant. TC: total cholesterol; FPG: fasting plasma glucose; TG: triglycerides; HDL: high-density lipoprotein; BMI: body mass index; WC: waist circumference; BP: blood pressure; WHR: waist-to-hip ratio; CVD: cardiovascular disease; PDM: pre-diabetes mellitus; OGTT: oral glucose tolerance test; Glu: glucose; HbA1c: glycated hemoglobin; SCR: serum creatinine; ALT: alanine transaminase; LDL: low-density lipoprotein; SBP: systolic blood pressure; DBP: diastolic blood pressure.

**Figure 3:**
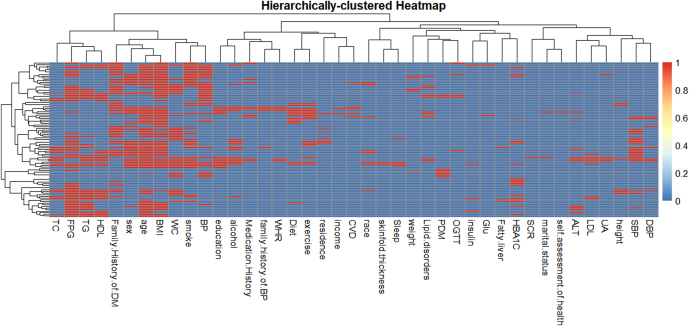
Hierarchically clustered heatmap. Heatmap of standardized predictor levels (columns) for all participants (rows). Ward linkage on Euclidean distance produced four predictor clusters (top dendrogram).

The co-occurrence network analysis of diabetes predictors, derived from hierarchical clustering of the binary dataset, revealed a multidimensional interplay among variables central to diabetes risk assessment. Core metabolic markers, including FPG, glucose levels (Glu), and TG, formed tightly interconnected clusters, emphasizing their established roles in glucose and lipid metabolism dysregulation, a hallmark of diabetes pathophysiology. HDL and waist-to-hip ratio (WHR) co-occurred within this metabolic cluster, suggesting their combined utility in identifying insulin resistance and adiposity-related risks (Figs. 2 and 3).

Anthropometric measures such as skinfold thickness, weight, and BMI exhibited strong associations with cardiovascular indicators (SBP, DBP, and family history of HBP), underscoring the synergistic contribution of obesity and hypertension to diabetes progression. Sociodemographic factors (education, marital status, and race) and lifestyle variables (alcohol, exercise, and sleep) clustered distinctly, reflecting their collective influence on behavioral and environmental risk stratification. The centrality of family history of diabetes mellitus (DM) and prediabetes mellitus (PDM) within the network highlights the critical role of genetic predisposition and early metabolic dysregulation in diabetes prediction (Figs 2 and 3).

This interconnectedness underscores the necessity of integrative models that account for biological, behavioral, and hereditary factors in diabetes prediction. The absence of isolated nodes (e.g., income, residence) further supports the hypothesis that socioeconomic and geographic determinants indirectly modulate diabetes risk through intermediate variables such as diet and healthcare access (17).

### Predictive model performance and validation

A total of 70.1% prediction models underwent training, 93.8% received internal validation, and 21.6% were externally validated (Supplementary Tables S1, S2 and S3). For training models, LR remained the dominant approach (82.4% of models), while ML algorithms, including XGBoost, RF, and Neural Network (NN), were applied in 16.2% of models. For internal validation, LR remained the dominant approach (73.6% of models), while ML algorithms, including XGBoost, RF, naïve Bayes (NB), decision trees (DT), and NN, were applied in 26.4% of models. In contrast, all externally validated studies exclusively relied on LR (85.7%) and Cox (14.3%), with no ML-based models undergoing external validation. Internal validation techniques (*n* = 91, 93.8% of models), including random data splitting (*n* = 38, 39.2% of models), cross-validation (*n* = 21, 21.6%), and bootstrapping (*n* = 18, 18.6% of models), were employed more frequently than external validation methods (*n* = 21, 21.6% of models), such as spatial validation (*n* = 12, 12.4% of models) or temporal validation (*n* = 8, 8.2%).

Evaluation of model discrimination using AUC-ROC or concordance index (C-index) revealed consistently moderate to high performance, with values spanning 0.628–0.998 across training, internal validation, and external validation. Calibration, assessed via Hosmer-Lemeshow (HL) tests or Brier scores in 57.7% of models, indicated adequate alignment between predicted and observed risks in most cases. Model presentation formats included risk score formulas (28.9%), nomograms (16.5%), and scoring sheets (13.4%), with limited adoption of dynamic or interactive tools. The predictive model performance and validation of the included studies are presented in Table 1.

### Risk of bias assessment using PROBAST tool

**Figure 4:**
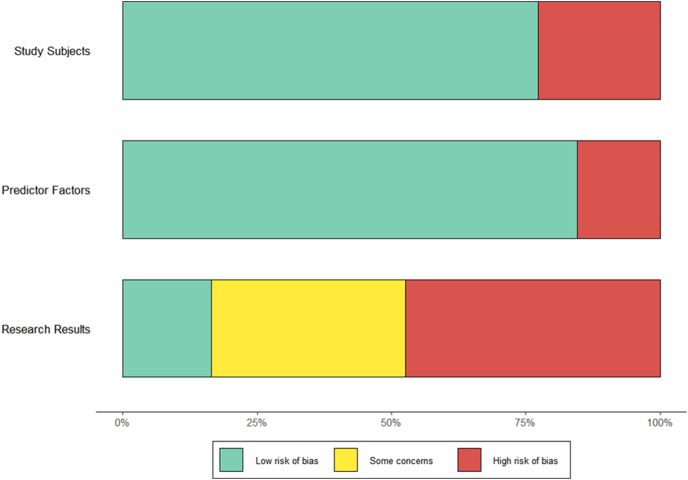
Risk of bias assessment for methodology.

**Figure 5:**
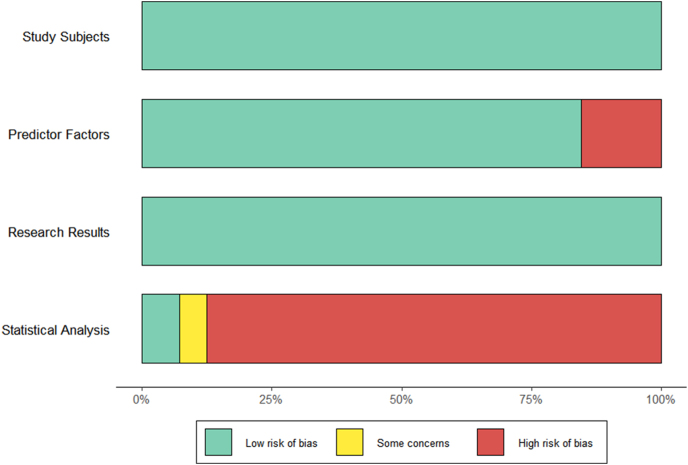
Risk of bias assessment for applicability.

The PROBAST-based evaluation of included studies (*n* = 65, encompassing 98 distinct prediction models) assessed the risk of bias across four domains: study subjects, predictor factors, research results, and statistical analysis, while also assessing the risk of applicability across three domains: study subjects, predictor factors, and research results (Table 2, Figs 4 and 5). For risk of bias, all studies demonstrated low risk in study subjects (100%), and most studies demonstrated low risk in predictor factors (84.5%), reflecting robust participant selection and clinically relevant predictor inclusion (e.g., age, BMI, FPG). However, 87.6% exhibited high risk in statistical analysis, primarily due to deletion of missing data or lack of external validation. Although external validation was performed, 5.2% of studies were rated as having unclear risk due to insufficient description of missing data handling. Overall, 91.8% of models were classified as high risk for bias. As for risk of applicability, the assessments of study subjects revealed low generalizability, with 15.5% rated low risk. In addition, 22.7% of studies were rated as high risk due to age-restricted cohorts. The evidence downgrading rationale for specific predictor factors corresponded to risk-of-bias assessments. Evidence quality was downgraded owing to either inappropriate handling of missing data (46.39%) or absence of documentation describing missing value processing procedures (34.02%).

**Table 2:** Risk of bias assessment.

Included studies	Study subjects	Predictor factors	Research results	Statistical analysis	Study subjects	Predictor factors	Research results	Risk of bias	Applicability
Han 2017 (23)	Low	Low	Low	High	High	Low	High	High	High
Sun 2013 (1) (21)	Low	Low	Low	Unclear	Low	Low	Unclear	High	High
Sun 2013 (2) (21)	Low	Low	Low	Unclear	Low	Low	Unclear	High	High
Sun 2013 (3) (21)	Low	Low	Low	Unclear	Low	Low	Unclear	High	High
Cai 2020 (24)	Low	High	Low	High	Low	High	Low	High	High
Hippisley-Cox 2009 (1) (18)	Low	Low	Low	Low	Low	Low	Low	Low	Low
Hippisley-Cox 2009 (2) (18)	Low	Low	Low	Low	Low	Low	Low	Low	Low
Wen 2016 (1) (25)	Low	Low	Low	High	Low	Low	Low	High	Low
Wen 2016 (2) (25)	Low	Low	Low	High	Low	Low	Low	High	Low
Wang 2018 (26)	Low	Low	Low	High	Low	Low	Low	High	Low
Bai 2018 (27)	Low	High	Low	High	Low	High	Unclear	High	High
Lei 2023 (28)	Low	High	Low	High	High	High	High	High	High
Fu 2023 (29)	Low	High	Low	High	High	High	High	High	High
Su 2017 (1) (30)	Low	High	Low	High	Low	High	Unclear	High	High
Su 2017 (2) (30)	Low	High	Low	High	Low	High	Unclear	High	High
Zhang 2022 (31)	Low	Low	Low	High	High	Low	Unclear	High	High
Yang 2016 (32)	Low	High	Low	High	Low	High	Unclear	High	High
Zhang 2016 (1) (33)	Low	Low	Low	High	Low	Low	Unclear	High	High
Zhang 2016 (2) (33)	Low	Low	Low	High	Low	Low	Unclear	High	High
Zhu 2023 (1) (34)	Low	Low	Low	High	Low	Low	High	High	High
Zhu 2023 (2) (34)	Low	Low	Low	High	Low	Low	High	High	High
Chen 2018 (35)	Low	Low	Low	High	Low	Low	Unclear	High	High
Ma 2020 (36)	Low	Low	Low	High	Low	Low	High	High	High
Liu 2020 (37)	Low	Low	Low	High	Low	Low	Low	High	Low
Lee 2024 (1) (38)	Low	Low	Low	Unclear	Low	Low	Unclear	High	High
Lee 2024 (2) (38)	Low	Low	Low	Unclear	Low	Low	Unclear	High	High
Alssema 2011 (39)	Low	Low	Low	High	High	Low	Unclear	High	High
Li 2019 (40)	Low	Low	Low	High	High	Low	High	High	High
Gao 2009 (1) (41)	Low	Low	Low	High	Low	Low	High	High	High
Gao 2009 (2) (41)	Low	Low	Low	High	Low	Low	High	High	High
Edlitz 2022 (1) (20)	Low	Low	Low	High	Low	Low	Low	High	Low
Edlitz 2022 (2) (20)	Low	Low	Low	High	Low	Low	Low	High	Low
Katsimpris 2021 (42)	Low	High	Low	High	Low	High	Unclear	High	High
Liu 2016 (43)	Low	Low	Low	High	High	Low	High	High	High
Hafezi 2024 (44)	Low	Low	Low	High	High	Low	Unclear	High	High
Hu 2020 (45)	Low	Low	Low	High	High	Low	Unclear	High	High
Xu 2014 (46)	Low	Low	Low	High	High	Low	Low	High	High
Rathmann 2010 (47)	Low	Low	Low	High	High	Low	Unclear	High	High
Liu 2022 (48)	Low	High	Low	High	High	High	Unclear	High	High
Zhang 2020 (49)	Low	High	Low	High	High	High	Unclear	High	High
Alghamdi 2017 (50)	Low	High	Low	High	Low	High	Unclear	High	High
Wang 2018 (1) (51)	Low	Low	Low	High	Low	Low	High	High	High
Wang 2018 (2) (51)	Low	Low	Low	High	Low	Low	High	High	High
Sun 2023 (52)	Low	Low	Low	High	Low	Low	Unclear	High	High
Stern 2002 (53)	Low	Low	Low	High	Low	Low	Unclear	High	High
Tran Quang 2022 (54)	Low	Low	Low	High	Low	Low	Unclear	High	High
Sun 2016 (55)	Low	Low	Low	High	High	Low	Unclear	High	High
Heianza 2012 (1) (56)	Low	Low	Low	High	Low	Low	High	High	High
Heianza 2012 (2) (56)	Low	Low	Low	High	Low	Low	High	High	High
Heianza 2012 (3) (56)	Low	Low	Low	High	Low	Low	High	High	High
Heianza 2012 (4) (56)	Low	Low	Low	High	Low	Low	High	High	High
Oh 2021 (1) (57)	Low	Low	Low	High	Low	Low	Unclear	High	High
Oh 2021 (2) (57)	Low	Low	Low	High	Low	Low	Unclear	High	High
Oh 2021 (3) (57)	Low	Low	Low	High	Low	Low	Unclear	High	High
Nicolaisen 2022 (58)	Low	Low	Low	High	Low	Low	High	High	High
Hu 2018 (1) (59)	Low	Low	Low	High	Low	Low	High	High	High
Hu 2018 (2) (59)	Low	Low	Low	High	Low	Low	High	High	High
Xu 2024 (1) (60)	Low	Low	Low	Low	Low	Low	Low	Low	Low
Xu 2024 (2) (60)	Low	Low	Low	Low	Low	Low	Low	Low	Low
Xu 2024 (3) (60)	Low	Low	Low	Low	Low	Low	Low	Low	Low
Arellano-Campos 2019 (61)	Low	Low	Low	High	Low	Low	Unclear	High	High
Shao 2020 (1) (62)	Low	Low	Low	High	Low	Low	High	High	High
Shao 2020 (2) (62)	Low	Low	Low	High	Low	Low	High	High	High
Shao 2020 (3) (62)	Low	Low	Low	High	Low	Low	High	High	High
Shao 2020 (4) (62)	Low	Low	Low	High	Low	Low	High	High	High
Rhee 2021 (63)	Low	Low	Low	High	Low	Low	Low	High	Low
Cai 2021 (64)	Low	Low	Low	High	Low	Low	Unclear	High	High
Olivera 2017 (65)	Low	Low	Low	High	Low	Low	High	High	High
Liu 2024 (66)	Low	Low	Low	High	Low	Low	High	High	High
Jagannathan 2020 (1) (67)	Low	Low	Low	High	Low	Low	High	High	High
Jagannathan 2020 (2) (67)	Low	Low	Low	High	Low	Low	High	High	High
Jagannathan 2020 (3) (67)	Low	Low	Low	High	Low	Low	High	High	High
Jagannathan 2020 (4) (67)	Low	Low	Low	High	Low	Low	High	High	High
Savolainen 2017 (68)	Low	Low	Low	High	Low	Low	Unclear	High	High
Sun 2009 (1) (69)	Low	Low	Low	High	Low	Low	High	High	High
Sun 2009 (2) (69)	Low	Low	Low	High	Low	Low	High	High	High
Lim 2012 (1) (70)	Low	Low	Low	High	Low	Low	High	High	High
Lim 2012 (2) (70)	Low	Low	Low	High	Low	Low	High	High	High
Lim 2012 (3) (70)	Low	Low	Low	High	Low	Low	High	High	High
Wang 2020 (71)	Low	High	Low	High	Low	High	High	High	High
Tan 2022 (72)	Low	High	Low	High	Low	High	High	High	High
Yatsuya 2018 (73)	Low	High	Low	High	Low	High	High	High	High
Wang 2022 (1) (74)	Low	Low	Low	High	High	Low	High	High	High
Wang 2022 (2) (74)	Low	Low	Low	High	High	Low	High	High	High
Wang 2022 (3) (74)	Low	Low	Low	High	High	Low	High	High	High
Wang 2022 (4) (74)	Low	Low	Low	High	High	Low	High	High	High
Wilson 2007 (75)	Low	Low	Low	High	High	Low	Unclear	High	High
Yan 2020 (19)	Low	Low	Low	High	Low	Low	High	Low	Low
Liu 2012 (1) (76)	Low	Low	Low	High	Low	Low	High	High	High
Liu 2012 (2) (76)	Low	Low	Low	High	Low	Low	High	High	High
Woo 2016 (77)	Low	Low	Low	High	High	Low	Unclear	High	High
Jiang 2023 (78)	Low	Low	Low	High	High	Low	High	High	High
Asgari 2021 (79)	Low	Low	Low	Low	Low	Low	Low	Low	Low
Stiglic 2021 (80)	Low	High	Low	High	High	High	Unclear	High	High
Lindström 2003 (81)	Low	Low	Low	High	Low	Low	High	High	High
Schmidt 2005 (82)	Low	Low	Low	High	Low	Low	Unclear	High	High
Chen 2010 (83)	Low	Low	Low	Low	Low	Low	Low	Low	Low

Low, low risk; High, high risk; Unclear, unclear risk.

### Subgroup analysis

#### Modeling

The subgroup analysis revealed variations in model performance across categories. Geographically, models from China (*n* = 38; AUC = 0.79, 95% CI: 0.77–0.81) and Japan (*n* = 12; AUC = 0.82, 95% CI: 0.78–0.86) demonstrated moderate discrimination, while USA-based models (*n* = 3; AUC = 0.97, 95% CI: 0.91–1.02) showed higher performance. By modeling approach, LR dominated (*n* = 56; AUC = 0.78, 95% CI: 0.76–0.80), whereas ML methods such as XGBoost (*n* = 3; AUC = 0.87, 95% CI: 0.76–0.99) and RF (*n* = 3; AUC = 0.86, 95% CI: 0.72–1.00) achieved higher AUCs than LR. Population-specific analysis indicated stronger performance in non-diabetic cohorts (*n* = 58; AUC = 0.80, 95% CI: 0.78–0.82) compared to prediabetic groups (*n* = 10; AUC = 0.74, 95% CI: 0.70–0.77). Follow-up duration (≥5 years: AUC = 0.81, 0.78–0.83), predictors (≥10: AUC = 0.80, 95% CI: 0.77–0.83), and sample size (≥1,000: AUC = 0.80, 95% CI: 0.77–0.82) showed better AUCs (Table 3).

**Table 3:** Subgroups of training.

Study	Numbers of predictive models	AUC (95% CI)	*I*^2^ (%)
**Total**	**68**	**0.79 (0.77–0.81)**	**99.5**
Country			
China	38	0.79 (0.77–0.81)	97.4
South Korea	4	0.75 (0.68–0.82)	97.1
Iran	3	0.77 (0.69–0.86)	98.0
USA	3	0.97 (0.91–1.02)	99.8
Japan	12	0.82 (0.78–0.86)	98.3
Sweden	1	0.64 (0.56–0.71)	–
Australia	1	0.78 (0.76–0.80)	–
Brazil	2	0.75 (0.74–0.76)	0
India	4	0.68 (0.65–0.72)	45.6
Machine			
LR	56	0.78 (0.76–0.80)	99.2
Cox	1	0.85 (0.82–0.88)	–
XGBoost	3	0.87 (0.76–0.99)	99.3
RF	3	0.86 (0.72–1.00)	99.6
NN	5	0.79 (0.77–0.82)	90.8
Population			
Non-diabetic	58	0.80 (0.78–0.82)	99.5
Prediabetic	10	0.74 (0.70–0.77)	87.5
Follow up (years)			
≥5	47	0.81 (0.78–0.83)	99.6
<5	21	0.76 (0.73–0.79)	93.3
Numbers			
≥1,000	54	0.80 (0.77–0.82)	99.5
<1,000	14	0.78 (0.72–0.83)	99.3
Number of predictors			
≥10	51	0.80 (0.77–0.83)	99.7
<10	17	0.78 (0.75–0.81)	96.1

LR, logistic regression; XGBoost, extreme gradient boosting; RF, random forest.

**Figure 6:**
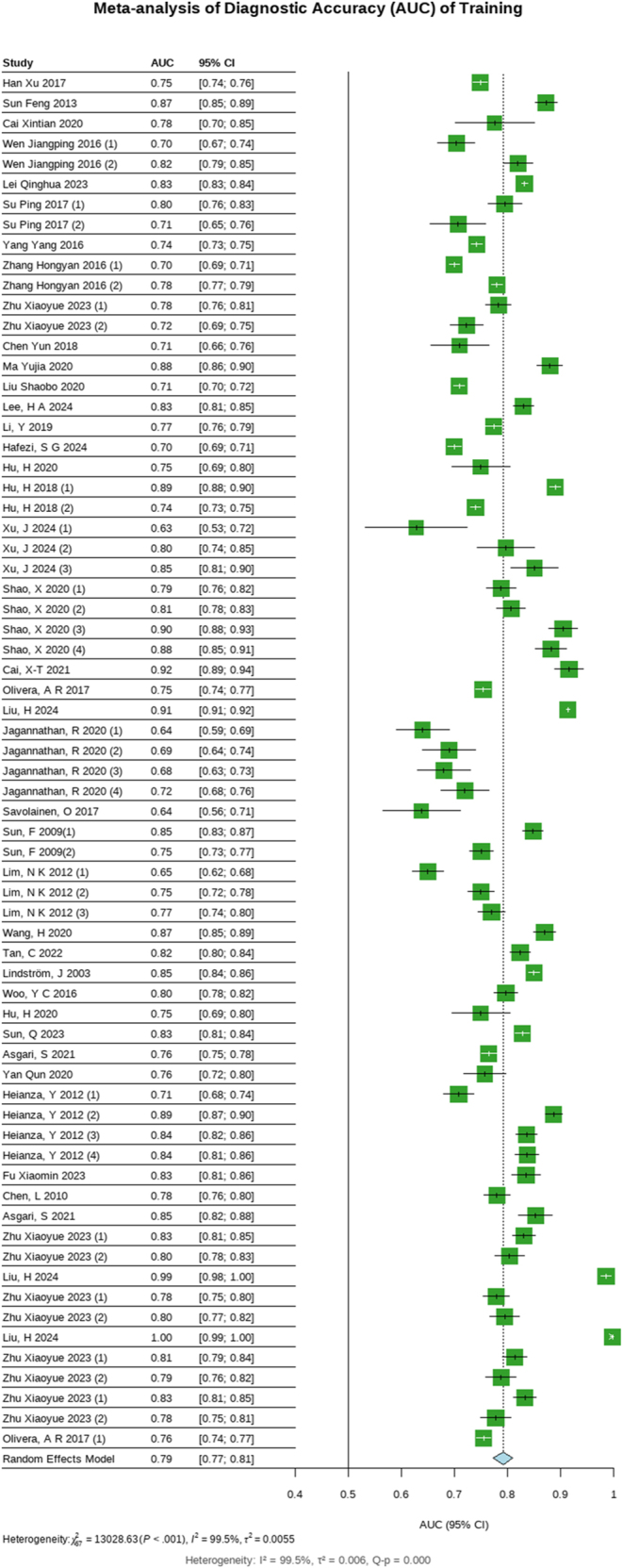
Meta-analysis of training.

**Figure 7:**
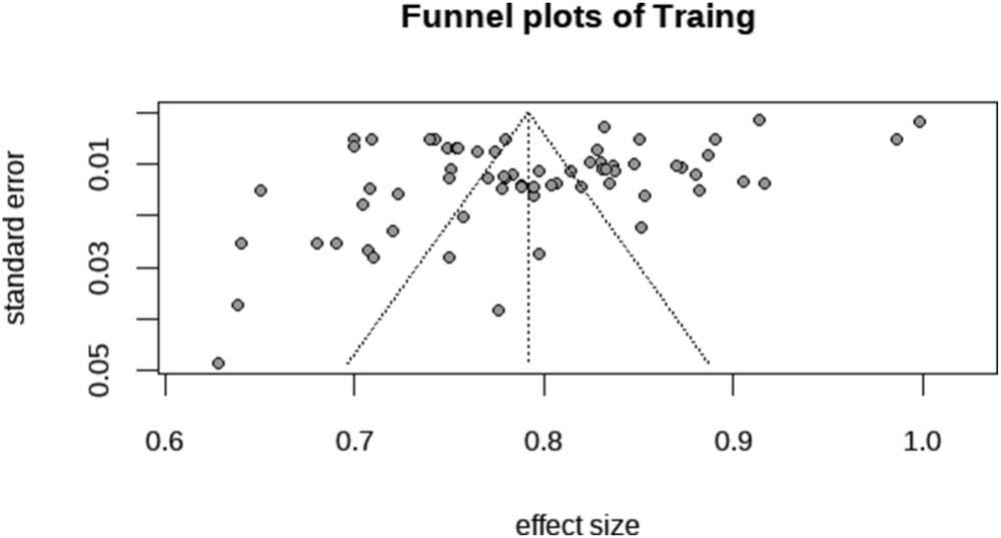
Funnel plots of training.

The results of sensitivity analysis demonstrated minimal variation in the effect estimates, with the 95% CIs remaining stable across all iterations (Supplementary Fig. 1). The overall results remained reliable, supporting the validity of the primary findings (Figs 6 and 7).

#### Internal validation

The subgroup analysis revealed distinct performance patterns across categories. Geographically, models from Germany (*n* = 2; AUC = 0.86, 95% CI: 0.81–0.91) and Japan (*n* = 8; AUC = 0.81, 95% CI: 0.75–0.87) demonstrated the highest discrimination, while Indian models (*n* = 3; AUC = 0.69, 95% CI: 0.66–0.72) showed the lowest. By modeling approach, LR dominated (*n* = 67; AUC = 0.78, 95% CI: 0.76–0.80), with RF (*n* = 8; AUC = 0.80, 95% CI: 0.74–0.86) achieving marginally higher performance. Population-specific analysis indicated stronger discrimination in non-diabetic cohorts (*n* = 79; AUC = 0.78, 95% CI: 0.76–0.80) compared to prediabetic groups (*n* = 9; AUC = 0.72, 95% CI: 0.71–0.73). Follow-up duration (≥5 years: AUC = 0.79, 95% CI: 0.77–0.82), (<10: AUC = 0.79, 95% CI: 0.77–0.81), and sample size (<1,000: AUC = 0.80, 95% CI: 0.76–0.84) showed a razor-thin differences (Table 4).

**Table 4:** Subgroups of internal validation.

Study	Numbers of predictive models	AUC (95% CI)	*I*^2^ (%)
**Total**	**91**	**0.78 (0.76–0.79)**	**99.9**
Country			
China	49	0.79 (0.77–0.81)	99.9
South Korea	7	0.76 (0.71–0.81)	96.5
Europe	2	0.73 (0.67–0.79)	97.6
USA	12	0.77 (0.72–0.82)	99.9
Germany	2	0.86 (0.81–0.91)	86.2
Iran	1	0.70 (0.68–0.71)	–
Australia	2	0.72 (0.66–0.77)	99%
Vietnam	1	0.71 (0.67–0.76)	–
Denmark	1	0.73 (0.71–0.74)	–
Japan	8	0.81 (0.75–0.87)	99.5
Mexico	1	0.75 (0.72–0.78)	–
Brazil	2	0.74 (0.74–0.76)	0
India	3	0.69 (0.66–0.72)	0
Machine			
LR	67	0.78 (0.76–0.80)	99.5
Cox	1	0.83 (0.82–0.83)	–
XGBoost	5	0.77 (0.71–0.83)	99.9
RF	8	0.80 (0.74–0.86)	99.9
NB	1	0.68 (0.67–0.68)	–
DT	2	0.68 (0.59–0.77)	99.8
NN	7	0.77 (0.73–0.80)	98.4
Population			
Non-diabetic	82	0.78 (0.77–0.80)	99.9
Prediabetic	9	0.72 (0.71–0.73)	30.1
Follow up (years)			
≥5	44	0.79 (0.77–0.82)	99.9
<5	47	0.76 (0.75–0.78)	99.1
Numbers			
≥1,000	75	0.77 (0.76–0.79)	99.9
<1,000	16	0.80 (0.76–0.84)	94.6
Number of predictors			
≥10	32	0.76 (0.73–0.79)	99.4
<10	59	0.79 (0.77–0.81)	99.9

LR, logistic regression; XGBoost, extreme gradient boosting; RF, random forest; NB, naïve Bayes; DT, decision tree.

**Figure 8:**
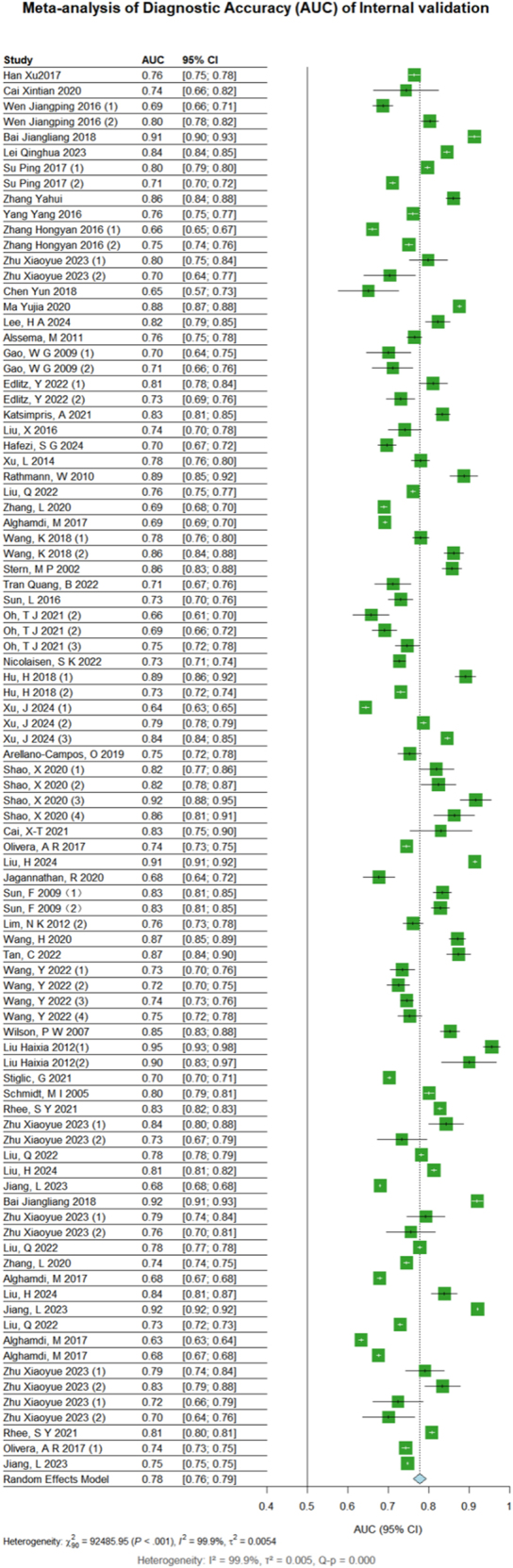
Meta-analysis of internal validation.

**Figure 9:**
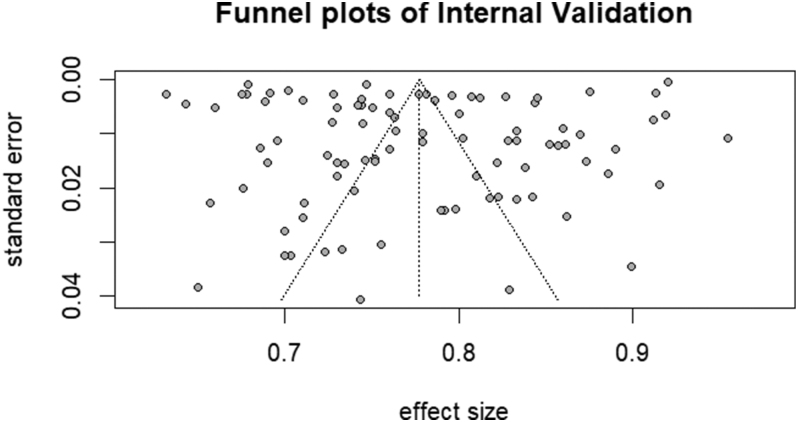
Full plots of internal validation.

The results of sensitivity analysis indicated minimal fluctuations in the effect estimates, with 95% CIs consistently overlapping across iterations (Supplementary Fig. 2). Funnel plot asymmetry was assessed using Egger’s regression test, which showed no significant evidence of publication bias (*P* = 0.0795). The symmetrical distribution of studies in the funnel plot further supported the absence of substantial bias (Figs 8 and 9).

#### External validation

Geographically, models from China and South Korea showed comparable discrimination. Japanese models (*n* = 7; AUC = 0.83, 95% CI: 0.76–0.89) displayed slightly lower performance. In addition, England models (*n* = 2; AUC = 0.84, 95% CI: 0.82–0.86) and the single Finnish model achieved higher AUC (0.87, 95% CI: 0.86–0.88). By sample size, models with ≥1,000 participants (*n* = 20; AUC = 0.81, 95% CI: 0.77–0.84) outperformed smaller cohorts (*n* = 1; AUC = 0.69, 95% CI: 0.65–0.74) (Table 5).

**Table 5:** Subgroups of external validation.

Study	Numbers of predictive models	AUC (95% CI)	*I*^2^ (%)
**Total**	**21**	**0.80 (0.73–0.87)**	**96.1**
Country			
China	4	0.83 (0.77–0.89)	98.6
South Korea	2	0.83 (0.81–0.85)	0
England	2	0.84 (0.82–0.86)	98.9
Japan	7	0.83 (0.76–0.89)	99.5
Finland	1	0.87 (0.86–0.88)	–
Iran	1	0.80 (0.79–0.81)	–
USA	2	0.68 (0.67–0.69)	15.4
Australia	2	0.72 (0.60–0.85)	87.8
Numbers			
≥1,000	20	0.81 (0.77–0.84)	99.3
<1,000	1	0.69 (0.63–0.74)	–

**Figure 10:**
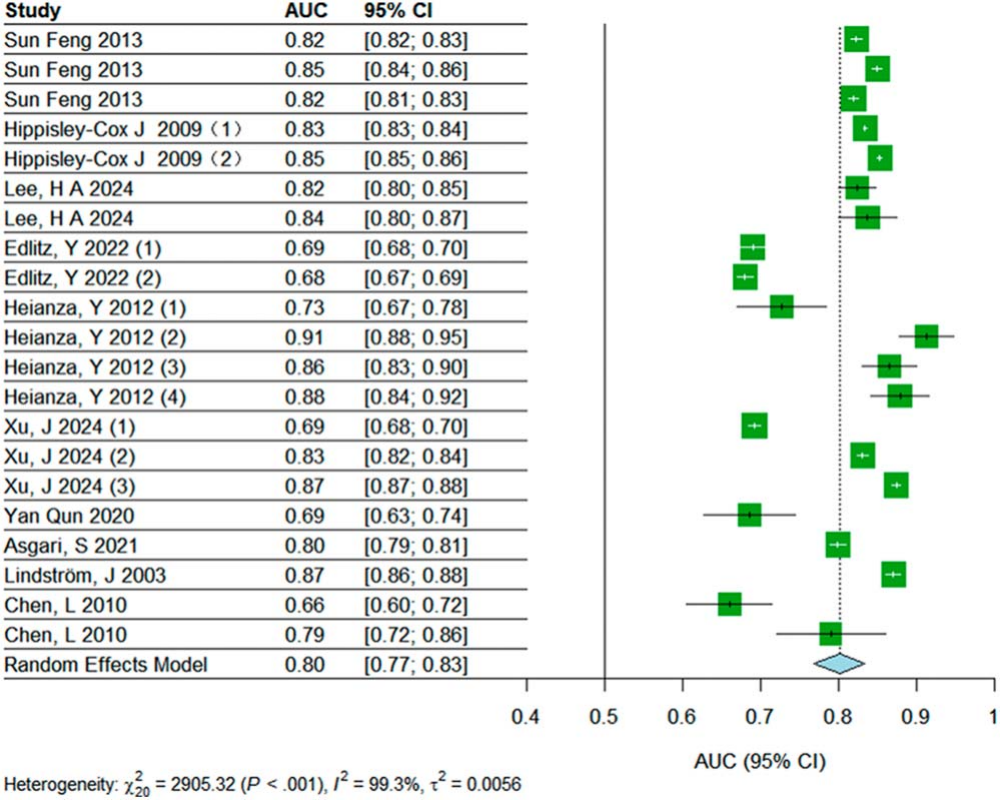
Meta-analysis of external validation.

**Figure 11:**
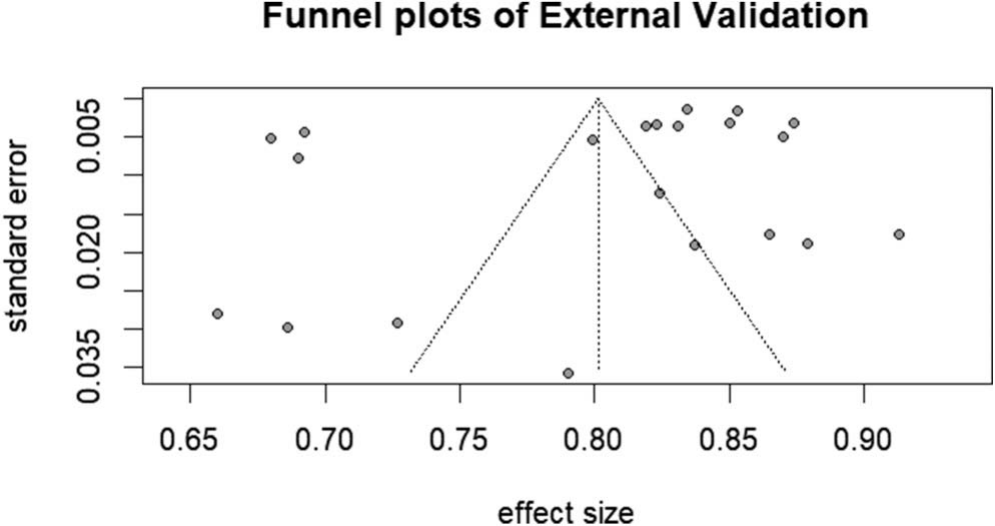
Funnel plots of external validation.

The results of sensitivity analysis demonstrated consistent effect sizes across iterations, with 95% CIs consistently overlapping across iterations (Supplementary Fig. 3). Egger’s regression test for funnel plot asymmetry revealed no statistically significant evidence of publication bias (*P* = 0.5701). The symmetrical distribution of studies in the funnel plot further supported the absence of substantial bias (Figs 10 and 11).

### Decision curve analysis

**Figure 12:**
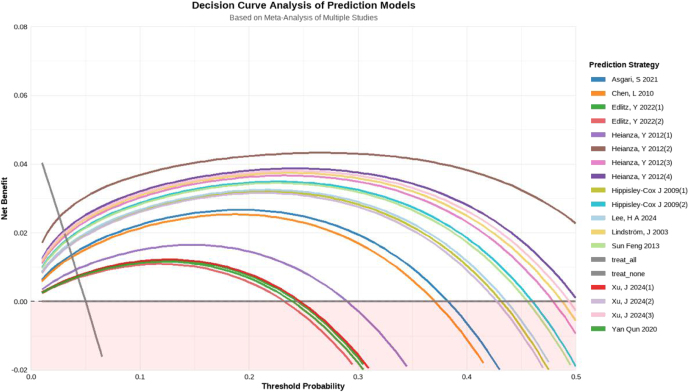
DCA. Net benefit curves derived from 19 pooled prediction models. The ‘treat-all’ (gray) and ‘treat-none’ (black) strategies serve as references.

DCA of 21 externally validated prediction models (Fig. 12) demonstrated substantial variation in clinical utility across threshold probabilities. Model performance exhibited threshold-dependent characteristics strongly associated with methodological and population factors. Models derived from larger cohorts (Hippisley-Cox *et al.* 2009 (1–2) (18): *n* = 1,232,832) maintained superior net benefit (>0.04 across 0.15–0.45 thresholds), while smaller studies (Yan *et al.* 2020 (19): *n* = 792; Chen *et al.* 2010 (83): *n* = 2,393–6,034) showed >30% variability at thresholds >0.4. Multi-predictor models (Heianza *et al.* 2012 (2) (56): seven predictors, AUC = 0.913; Heianza *et al.* 2012 (4) (56): six predictors, AUC = 0.879) achieved sustained net benefit (>0.03) over broader threshold ranges (0.2–0.4), contrasting with simpler constructs (Xu *et al.* 2024 (1) (60): two predictors, AUC = 0.692) that demonstrated threshold-specific limitations. East Asian cohorts (Xu *et al.* 2024 (3) (60): Japan, AUC = 0.874) peaked at 0.25–0.35 thresholds (net benefit >0.05). Western populations (Edlitz & Segal 2022 (1–2) (20): USA, AUC: 0.68–0.69) showed optimal performance at higher thresholds (>0.35). The English cohort (Hippisley-Cox *et al.* 2009 (2) (18): AUC = 0.853) demonstrated exceptional generalizability. The highest-AUC model (Heianza *et al.* 2012 (2) (56): AUC = 0.913) showed threshold-restricted utility (0.2–0.3), while moderate-AUC models with larger samples (Sun *et al.* 2013 (21), AUC: 0.819–0.85, *n* = 12,392) maintained >0.035 net benefit across 0.15–0.4 thresholds.

## Discussion

The escalating global prevalence of T2DM underscores the urgent need for robust risk prediction models to guide early intervention and precision prevention strategies (22, 23). This systematic review and meta-analysis critically appraise 65 multivariable prediction studies (encompassing 97 distinct prediction models) published between 2002 and 2024. The pooled discrimination metrics (AUC: 0.628–0.998) align with prior systematic reviews, which reported similar ranges but highlighted inconsistent validation practices (7). Notably, ML models achieved marginally higher AUCs than LR (e.g., training, XGBoost AUC: 0.87 vs LR: 0.78; internal validation, RF AUC: 0.80 vs LR AUC: 0.78), yet their real-world implementation is hampered by limited interpretability and lack of external validation (21.6% of externally validated models used LR) (4). The challenges identified in the PROBAST framework, where 91.8% of models exhibited high risks of bias due to inadequate statistical reporting and lack of external verification, and 84.5% of models exhibited high risks of applicability due to inadequate statistical reporting (e.g., missing data deletion without justification) and insufficient adjustment for confounders (5). We urge journals to mandate adherence to the TRIPOD-AI statement (22) for transparent reporting of prediction models, especially for ML-based studies.

Predictor network analysis revealed metabolic markers (FPG, TG, HDL) and anthropometric measures (BMI, WC) as interconnected hubs, while socioeconomic factors (education, income) acted through indirect pathways (e.g., diet access). This supports developing integrated models that combine clinical, behavioral, and social determinants.

Geographic disparities (USA AUC: 0.97 vs China AUC: 0.79) and poor performance in pre-diabetic cohorts (AUC: 0.72 vs 0.80 in normoglycemic) highlight the need for context-specific adaptations. To address these gaps, we recommend prioritizing external validation across diverse populations, particularly for ML models, of which only 21.6% have undergone any form of external validation. Furthermore, we propose the establishment of multinational consortia to facilitate the sharing of harmonized data for the purposes of model training and testing. In addition, we advocate for the development of dynamic prediction tools that integrate time-varying predictors, such as lifestyle changes, with a particular focus on the progression of prediabetes.

The study has some limitations, including language bias (only Chinese/English studies) and outcome definition variability. The inclusion of studies published in Chinese may limit the ability of the international scientific community to independently assess the full details of these studies, potentially affecting the reproducibility and critical appraisal of those specific models by non-Chinese readers. We advocate that researchers, especially in regions producing high volumes of research, register study protocols and share model specifications (e.g., coefficients, nomograms, code) on international platforms (e.g., ClinicalTrials.gov, DRYAD, Figshare), which would significantly enhance global accessibility and reproducibility, regardless of the primary publication language. Second, although we requested calibration intercept and slope from authors and searched appendices, these metrics were unavailable for the vast majority of models. We, therefore, present a qualitative summary of the calibration plots. This limitation reinforces our recommendation that future prediction-model studies adopt the TRIPOD-AI statement, which mandates calibration intercept, slope, and full calibration plots.

In conclusion, while T2DM prediction models show promise, their clinical utility is compromised by methodological shortcomings and limited generalizability. By implementing structured reporting standards, fostering cross-border collaboration, and focusing on high-risk subgroups, future models can advance precision prevention and mitigate the global diabetes burden.

## Supplementary materials



## Declaration of interest

The authors declare that there is no conflict of interest that could be perceived as prejudicing the impartiality of the work reported.

## Funding

This research was funded by the State Key Laboratory of Dampness Syndrome of Chinese Medicine (No. 2022KT1315, 2020KT1074), the National Key Technology R&D Program for the 13th Five-year Plan of the Ministry of Science and Technology, China (No. 2018YFC1707407), and the Guangdong Provincial Hospital of Chinese Medicine Special Project for Science and Technology Research in Traditional Chinese Medicine (Grant No. YN2020QN28).

## Author contribution statement

Meichen Li designed the study. Linjie Song and Xingying Qiu conducted the study. Meichen Li and Aowei Tan performed the data analysis. Meichen Li and Aowei Tan interpreted the data. All authors drafted the manuscript. Zhou Li and Zeihuai Wen reviewed and edited the manuscript. Zehuai Wen acquired the funding. All authors approved the final version of the manuscript.
