# Supplementary material for: Performance of diabetes risk prediction models: a systematic review and meta-analysis
Source: Endocr Connect. 2025 Nov 3;14(11):e250353. doi: 10.1530/EC-25-0353 (PMC12586975; doi:10.1530/EC-25-0353)
Supplement: Supplementary file 1 [file supplementary_materials.pdf]

Table S1 Characteristics of Model Development Studies of Training

Model	Study	year	Predictors	AUC	Lower_CI	Upper_CI	Numbers	Country	Population
LR	Han Xu 2017	5	7	0.749	0.735	0.762	17690	China	Non-diabetic
LR	Sun Feng 2013	5	8	0.873	0.851	0.893	12392	China	Non-diabetic
LR	Cai Xintian 2020	5	8	0.776	0.699	0.849	22936	China	Non-diabetic
LR	Wen Jiangping 2016 (1)	6	4	0.704	0.669	0.739	2754	China	Non-diabetic
LR	Wen Jiangping 2016 (2)	6	6	0.820	0.791	0.848	4132	China	Non-diabetic
LR	Lei Qinghua 2023	5	6	0.832	--	--	17363	China	Non-diabetic
LR	Su Ping 2017 (1)	3	6	0.795	0.764	0.827	18963	China	Non-diabetic
LR	Su Ping 2017 (2)	3	5	0.707	0.654	0.759	14482	China	Non-diabetic
LR	Yang Yang 2016	5	6	0.742	0.732	0.752	16715	China	Non-diabetic
LR	Zhang Hongyan 2016 (1)	6	12	0.700	0.690	0.710	6143	China	Non-diabetic
LR	Zhang Hongyan 2016 (2)	6	5	0.780	0.770	0.790	6143	China	Non-diabetic
LR	Zhu Xiaoyue 2023 (1)	3	37	0.783	--	--	1133	China	Non-diabetic
LR	Zhu Xiaoyue 2023 (2)	3	33	0.723	--	--	796	China	Prediabetic
LR	Chen Yun 2018	1	4	0.710	0.660	0.770	3803	China	Prediabetic
LR	Ma Yujia 2020	2	6	0.880	0.856	0.903	3127	China	Non-diabetic
LR	Liu Shaobo 2020	6	7	0.709	0.701	0.722	7289	China	Non-diabetic
LR	Lee, H A 2024	8	8	0.830	0.811	0.849	5459	South Korea	Non-diabetic
LR	Li, Y 2019	5	10	0.774	0.759	0.789	17173	China	Non-diabetic
LR	Hafezi, S G 2024	10	3	0.6998	--	--	5033	Iran	Non-diabetic
LR	Hu, H 2020	5	7	0.750	0.700	0.810	4833	China	Non-diabetic
LR	Hu, H 2018 (1)	7	9	0.890	0.880	0.900	30500	Japan	Non-diabetic
LR	Hu, H 2018 (2)	7	6	0.740	0.730	0.750	30500	Japan	Non-diabetic
LR	Xu, J 2024 (1)	5	4	0.628	0.532	0.723	10986	Japan	Non-diabetic
LR	Xu, J 2024 (2)	5	2	0.797	0.743	0.851	10986	Japan	Non-diabetic
LR	Xu, J 2024 (3)	5	4	0.851	0.807	0.895	10986	Japan	Non-diabetic
LR	Shao, X 2020 (1)	10	8	0.788	0.761	0.816	4498	China	Non-diabetic
LR	Shao, X 2020 (2)	10	13	0.807	0.780	0.834	4498	China	Non-diabetic
LR	Shao, X 2020 (3)	10	20	0.905	0.879	0.932	4498	China	Non-diabetic
LR	Shao, X 2020 (4)	10	7	0.882	0.853	0.912	4498	China	Non-diabetic
LR	Cai, X-T 2021	5	6	0.916	0.889	0.943	9651	Japan	Non-diabetic
LR	Olivera, A R 2017	10	13	0.754	--	--	3709	Brazil	Non-diabetic
LR	Liu, H 2024	5	8	0.914	--	--	32372	USA	Non-diabetic
LR	Jagannathan, R 2020 (1)	3	9	0.640	0.590	0.690	548	India	Prediabetic
LR	Jagannathan, R 2020 (2)	3	10	0.690	0.640	0.740	548	India	Prediabetic
LR	Jagannathan, R 2020 (3)	3	11	0.680	0.630	0.730	548	India	Prediabetic
LR	Jagannathan, R 2020 (4)	3	12	0.720	0.670	0.760	548	India	Prediabetic
LR	Savolainen, O 2017	5	5	0.638	0.565	0.711	629	Sweden	Non-diabetic
LR	Sun, F 2009(1)	5	7	0.848	0.829	0.868	10294	China	Non-diabetic

LR	Sun, F 2009(2)	5	6	0.751	0.730	0.773	10294	China	Non-diabetic
LR	Lim, N K 2012 (1)	4	5	0.650	0.620	0.680	6342	South Korea	Non-diabetic
LR	Lim, N K 2012 (2)	4	8	0.750	0.720	0.770	6342	South Korea	Non-diabetic
LR	Lim, N K 2012 (3)	4	9	0.770	0.740	0.790	6342	South Korea	Non-diabetic
LR	Wang, H 2020	5	6	0.870	0.850	0.890	8296	Japan	Non-diabetic
LR	Tan, C 2022	6	6	0.824	--	--	1518	Japan	Non-diabetic
LR	Lindström, J 2003	10	7	0.850	--	--	4746	China	Non-diabetic
LR	Woo, Y C 2016	9	6	0.797	0.773	0.817	1380	China	Non-diabetic
LR	Hu, H 2020	5	7	0.750	0.700	0.810	4833	China	Non-diabetic
LR	Sun, Q 2023	7	9	0.828	--	--	2755	China	Non-diabetic
LR	Asgari, S 2021	9	8	0.765	--	--	3147	Iran	Non-diabetic
LR	Yan Qun 2020	5	4	0.757	0.723	0.802	810	China	Non-diabetic
LR	Heianza, Y 2012 (1)	5	5	0.708	0.679	0.737	7654	Japan	Non-diabetic
LR	Heianza, Y 2012 (2)	5	7	0.887	0.871	0.903	7654	Japan	Non-diabetic
LR	Heianza, Y 2012 (3)	5	6	0.836	0.815	0.856	7654	Japan	Non-diabetic
LR	Heianza, Y 2012 (4)	5	6	0.837	0.815	0.859	7654	Japan	Non-diabetic
LR	Fu Xiaomin 2023	7	7	0.835	--	--	712	China	Non-diabetic
LR	Chen, L 2010	5	9	0.78	0.76	0.81	6060	Australia	Non-diabetic
COX	Asgari, S 2021	9	8	0.853	0.822	0.885	7654	Iran	Non-diabetic
XGBOOST	Zhu Xiaoyue 2023 (1)	3	37	0.831	--	--	1133	China	Non-diabetic
XGBOOST	Zhu Xiaoyue 2023 (2)	3	33	0.804	--	--	796	China	Prediabetic
XGBOOST	Liu, H 2024	5	8	0.986	--	--	518	USA	Non-diabetic
RF	Zhu Xiaoyue 2023 (1)	3	37	0.779	--	--	1133	China	Non-diabetic
RF	Zhu Xiaoyue 2023 (2)	3	33	0.795	--	--	796	China	Prediabetic
RF	Liu, H 2024	5	8	0.998	--	--	518	USA	Non-diabetic
FNN	Zhu Xiaoyue 2023 (1)	3	37	0.814	--	--	1133	China	Non-diabetic
FNN	Zhu Xiaoyue 2023 (2)	3	33	0.788	--	--	796	China	Prediabetic
CNN	Zhu Xiaoyue 2023 (1)	3	37	0.833	--	--	1133	China	Non-diabetic
CNN	Zhu Xiaoyue 2023 (2)	3	33	0.778	--	--	796	China	Prediabetic
ANN	Olivera, A R 2017 (1)	10	13	0.755	--	--	3709	Brazil	Non-diabetic

Table S2 Characteristics of Model Development Studies of Internal validation

Model	Study	year	Predictors	AUC	Lower_CI	Upper_CI	Numbers	Country	Population
LR	Han Xu 2017	5	7	0.763	0.749	0.776	17690	China	Non-diabetic
LR	Cai Xintian 2020	5	8	0.743	0.665	0.824	9830	China	Non-diabetic
LR	Wen Jiangping 2016 (1)	6	4	0.686	0.661	0.71	1378	China	Non-diabetic
LR	Wen Jiangping 2016 (2)	6	6	0.802	0.78	0.822	1378	China	Non-diabetic
LR	Bai Jiangliang 2018	5	14	0.912	0.898	0.927	3531	China	Non-diabetic
LR	Lei Qinghua 2023	5	6	0.844	--	--	7441	China	Non-diabetic
LR	Su Ping 2017 (1)	3	6	0.796	--	--	18963	China	Non-diabetic
LR	Su Ping 2017 (2)	3	5	0.710	--	--	14482	China	Non-diabetic
LR	Zhang Yahui	3	11	0.860	--	--	1512	China	Non-diabetic
LR	Yang Yang 2016	5	6	0.760	0.748	0.772	16715	China	Non-diabetic
LR	Zhang Hongyan 2016 (1)	6	12	0.660	0.650	0.670	6142	China	Non-diabetic
LR	Zhang Hongyan 2016 (2)	6	5	0.750	0.740	0.760	6142	China	Non-diabetic
LR	Zhu Xiaoyue 2023 (1)	3	37	0.798	--	--	284	China	Non-diabetic
LR	Zhu Xiaoyue 2023 (2)	3	33	0.703	--	--	199	China	Prediabetic
LR	Chen Yun 2018	1	5	0.65	0.58	0.73	1853	China	Prediabetic
LR	Ma Yujia 2020	2	6	0.875	0.870	0.879	3127	China	Non-diabetic
LR	Lee, H A 2024	8	8	0.822	0.792	0.852	2318	South Korea	Non-diabetic
LR	Alssema, M 2011	5	8	0.764	0.746	0.783	18301	Europe	Non-diabetic
LR	Gao, W G 2009 (1)	10	7	0.700	0.640	0.750	1182	India	Non-diabetic
LR	Gao, W G 2009 (2)	10	7	0.710	0.660	0.760	1550	India	Non-diabetic
LR	Edlitz, Y 2022 (1)	5	7	0.810	0.770	0.840	7948	USA	Non-diabetic
LR	Edlitz, Y 2022 (2)	5	7	0.730	0.690	0.760	1006	USA	Prediabetic
LR	Katsimpris, A 2021	5	4	0.833	--	--	1591	Germany	Non-diabetic
LR	Liu, X 2016	20	11	0.740	0.700	0.78	1857	China	Non-diabetic
LR	Hafezi, S G 2024	10	3	0.6958	--	--	1677	Iran	Non-diabetic
LR	Xu, L 2014	4	6	0.779	0.756	0.801	8043	China	Non-diabetic
LR	Rathmann, W 2010	7	10	0.886	0.852	0.92	887	Germany	Non-diabetic
LR	Liu, Q 2022	2	21	0.760	--	--	25406	China	Non-diabetic
LR	Zhang, L 2020	10	13	0.6885	0.6801	0.6961	70975	Australia	Non-diabetic
LR	Alghamdi, M 2017	5	11	0.691	--	--	32555	USA	Non-diabetic
LR	Wang, K 2018 (1)	3	9	0.779	0.76	0.799	5087	China	Non-diabetic
LR	Wang, K 2018 (2)	3	9	0.861	0.838	0.885	3549	China	Non-diabetic
LR	Stern, M P 2002	7	9	0.857	0.834	0.882	2903	USA	Non-diabetic
LR	Tran Quang, B 2022	5	5	0.711	0.666	0.755	1448	Vietnam	Non-diabetic
LR	Sun, L 2016	6	11	0.730	0.700	0.760	2103	China	Non-diabetic
LR	Oh, T J 2021 (2)	10	5	0.657	0.626	0.715	1700	South Korea	Non-diabetic
LR	Oh, T J 2021 (2)	10	6	0.690	0.66	0.720	1700	South	Non-diabetic

								Korea	
LR	Oh, T J 2021 (3)	10	6	0.746	0.717	0.775	1700	South Korea	Non-diabetic
LR	Nicolaisen, S K 2022	5	7	0.727	0.712	0.743	5201	Denmark	Prediabetic
LR	Hu, H 2018 (1)	7	9	0.890	0.870	0.920	13349	Japan	Non-diabetic
LR	Hu, H 2018 (2)	7	6	0.730	0.720	0.740	13349	Japan	Non-diabetic
LR	Xu, J 2024 (1)	5	4	0.643	--	--	10986	Japan	Non-diabetic
LR	Xu, J 2024 (2)	5	2	0.786	--	--	10986	Japan	Non-diabetic
LR	Xu, J 2024 (3)	5	3	0.845	--	--	10986	Japan	Non-diabetic
LR	Arellano-Campos, O 2019	3	5	0.752	0.724	0.781	6144	Mexico	Non-diabetic
LR	Shao, X 2020 (1)	10	8	0.818	0.775	0.861	1525	China	Non-diabetic
LR	Shao, X 2020 (2)	10	13	0.823	0.780	0.865	1525	China	Non-diabetic
LR	Shao, X 2020 (3)	10	20	0.915	0.877	0.953	1525	China	Non-diabetic
LR	Shao, X 2020 (4)	10	7	0.862	0.813	0.912	1525	China	Non-diabetic
LR	Cai, X-T 2021	5	6	0.829	0.753	0.905	3289	Japan	Non-diabetic
LR	Olivera, A R 2017	10	13	0.744	--	--	8738	Brazil	Non-diabetic
LR	Liu, H 2024	5	8	0.913	--	--	13875	USA	Non-diabetic
LR	Jagannathan, R 2020	3	11	0.676	--	--	548	India	Prediabetic
LR	Sun, F 2009（1）	5	9	0.833	0.811	0.855	10257	China	Non-diabetic
LR	Sun, F 2009（2）	5	8	0.828	0.807	0.851	10257	China	Non-diabetic
LR	Lim, N K 2012 (2)	4	9	0.760	0.730	0.780	6342	South Korea	Non-diabetic
LR	Wang, H 2020	5	6	0.870	0.860	0.900	2817	Japan	Non-diabetic
LR	Tan, C 2022	6	6	0.873	--	--	494	Japan	Non-diabetic
LR	Wang, Y 2022 (1)	4	3	0.734	0.703	0.764	15934	China	Non-diabetic
LR	Wang, Y 2022 (2)	4	3	0.724	0.697	0.752	4324	China	Non-diabetic
LR	Wang, Y 2022 (3)	4	3	0.745	0.729	0.761	11610	China	Non-diabetic
LR	Wang, Y 2022 (4)	4	9	0.752	0.723	0.782	15934	China	Non-diabetic
LR	Wilson, P W 2007	8	10	0.852	0.828	0.875	3140	USA	Non-diabetic
LR	Liu Haixia 2012(1)	5	7	0.955	0.934	0.976	596	China	Non-diabetic
LR	Liu Haixia 2012(2)	5	7	0.899	0.832	0.967	487	China	Non-diabetic
LR	Stiglic, G 2021	10	5	0.702	0.698	0.706	16363	Europe	Non-diabetic
LR	Schmidt, M I 2005	9	13	0.8	--	--	3957	USA	Non-diabetic
COX	Rhee, S Y 2021	10	10	0.827	0.821	0.833	67061	South Korea	Non-diabetic
XGBOOST	Zhu Xiaoyue 2023 (1)	3	37	0.842	--	--	284	China	Non-diabetic
XGBOOST	Zhu Xiaoyue 2023 (2)	3	33	0.733	--	--	199	China	Prediabetic
XGBOOST	Liu, Q 2022	2	21	0.781	--	--	25406	China	Non-diabetic
XGBOOS	Liu, H 2024	5	8	0.812	--	--	13875	USA	Non-diabetic

T									
XGBOOST	Jiang, L 2023	7	7	0.679	--	--	252176	China	Non-diabetic
RF IV AUC	Bai Jiangliang 2018	5	14	0.919	0.906	0.932	3531	China	Non-diabetic
RF	Zhu Xiaoyue 2023 (1)	3	37	0.792	--	--	284	China	Non-diabetic
RF	Zhu Xiaoyue 2023 (2)	3	33	0.755	--	--	199	China	Prediabetic
RF	Liu, Q 2022	2	21	0.777	--	--	25406	China	Non-diabetic
RF	Zhang, L 2020	10	15	0.7439	0.7365	0.751	236684	Australia	Non-diabetic
RF	Alghamdi, M 2017	5	11	0.678	--	--	32555	USA	Non-diabetic
RF	Liu, H 2024	5	8	0.838	--	--	518	USA	Non-diabetic
RF	Jiang, L 2023	7	7	0.92	--	--	252176	China	Non-diabetic
DT	Liu, Q 2022	2	21	0.728	--	--	25406	China	Non-diabetic
DT	Alghamdi, M 2017	5	11	0.632	--	--	32555	USA	Non-diabetic
NB	Alghamdi, M 2017	5	11	0.675	--	--	32555	USA	Non-diabetic
FNN	Zhu Xiaoyue 2023 (1)	3	37	0.790	--	--	284	China	Non-diabetic
CNN	Zhu Xiaoyue 2023 (2)	3	33	0.833	--	--	284	China	Non-diabetic
FNN	Zhu Xiaoyue 2023 (1)	3	37	0.723	--	--	199	China	Prediabetic
CNN	Zhu Xiaoyue 2023 (2)	3	33	0.700	--	--	199	China	Prediabetic
RNN	Rhee, S Y 2021	10	10	0.807	0.801	0.813	67061	South Korea	Non-diabetic
ANN IV AUC	Olivera, A R 2017 (1)	10	13	0.742	--	--	8738	Brazil	Non-diabetic
KNN	Jiang, L 2023	7	7	0.747	--	--	252176	China	Non-diabetic

Table S3 Characteristics of Model Development Studies of External validation

Model	Study	year	Predictors	AUC	Lower_CI	Upper_CI	Numbers	Country	Population
LR	Sun Feng 2013	5	8	0.823	--	--	12392	China	Non-diabetic
LR	Sun Feng 2013	5	8	0.850	--	--	12392	China	Non-diabetic
LR	Sun Feng 2013	5	8	0.819	--	--	12392	China	Non-diabetic
Cox	Hippisley-Cox J 2009 （1）	10	10	0.834	0.831	0.836	1232832	England	Non-diabetic
Cox	Hippisley-Cox J 2009 （2）	10	10	0.853	0.850	0.856	1232832	England	Non-diabetic
LR	Lee, H A 2024	8	8	0.824	0.8	0.848	2043	South Korea	Non-diabetic
LR	Lee, H A 2024	8	8	0.837	0.8	0.875	4091	South Korea	Non-diabetic
LR	Edlitz, Y 2022 (1)	5	7	0.69	0.66	0.69	10064	USA	Non-diabetic
LR	Edlitz, Y 2022 (2)	5	7	0.68	0.67	0.69	7059	USA	Prediabetic
LR	Heianza, Y 2012 (1)	5	5	0.727	0.67	0.784	1976	Japan	Non-diabetic
LR	Heianza, Y 2012 (2)	5	7	0.913	0.878	0.947	1976	Japan	Non-diabetic
LR	Heianza, Y 2012 (3)	5	6	0.865	0.831	0.9	1976	Japan	Non-diabetic
LR	Heianza, Y 2012 (4)	5	6	0.879	0.842	0.916	1976	Japan	Non-diabetic
LR	Xu, J 2024 (1)	5	2	0.692	--	--	11345	Japan	Non-diabetic
LR	Xu, J 2024 (2)	5	3	0.831	--	--	11345	Japan	Non-diabetic
LR	Xu, J 2024 (3)	5	4	0.874	--	--	11345	Japan	Non-diabetic
LR	Yan Qun 2020	5	4	0.686	0.628	0.745	792	China	Non-diabetic
LR	Lindström, J 2003	10	7	0.87	--	--	4746	Finland	Non-diabetic
COX	Asgari, S 2021	9	8	0.799	0.789	0.810	3147	Iran	Non-diabetic
LR	Chen, L 2010	5	9	0.660	0.60	0.710	2393	Australia	Non-diabetic
LR	Chen, L 2010	5	9	0.790	0.720	0.860	6034	Australia	Non-diabetic

Sensitivity Analysis of Modling

Omitted Study	AUC	95% CI
Omitting Han Xu 2017	0.79	[0.77; 0.81]
Omitting Sun Feng 2013	0.79	[0.77; 0.81]
Omitting Cai Xintian 2020	0.79	[0.77; 0.81]
Omitting Wen Jiangping 2016 (1)	0.79	[0.77; 0.81]
Omitting Wen Jiangping 2016 (2)	0.79	[0.77; 0.81]
Omitting Lei Qinghua 2023	0.79	[0.77; 0.81]
Omitting Su Ping 2017 (1)	0.79	[0.77; 0.81]
Omitting Su Ping 2017 (2)	0.79	[0.77; 0.81]
Omitting Yang Yang 2016	0.79	[0.77; 0.81]
Omitting Zhang Hongyan 2016 (1)	0.79	[0.77; 0.81]
Omitting Zhang Hongyan 2016 (2)	0.79	[0.77; 0.81]
Omitting Zhu Xiaoyue 2023 (1)	0.79	[0.77; 0.81]
Omitting Zhu Xiaoyue 2023 (2)	0.79	[0.77; 0.81]
Omitting Chen Yun 2018	0.79	[0.77; 0.81]
Omitting Ma Yujia 2020	0.79	[0.77; 0.81]
Omitting Liu Shaobo 2020	0.79	[0.77; 0.81]
Omitting Lee, H A 2024	0.79	[0.77; 0.81]
Omitting Li, Y 2019	0.79	[0.77; 0.81]
Omitting Hafezi, S G 2024	0.79	[0.77; 0.81]
Omitting Hu, H 2020	0.79	[0.77; 0.81]
Omitting Hu, H 2018 (1)	0.79	[0.77; 0.81]
Omitting Hu, H 2018 (2)	0.79	[0.77; 0.81]
Omitting Xu, J 2024 (1)	0.79	[0.78; 0.81]
Omitting Xu, J 2024 (2)	0.79	[0.77; 0.81]
Omitting Xu, J 2024 (3)	0.79	[0.77; 0.81]
Omitting Shao, X 2020 (1)	0.79	[0.77; 0.81]
Omitting Shao, X 2020 (2)	0.79	[0.77; 0.81]
Omitting Shao, X 2020 (3)	0.79	[0.77; 0.81]
Omitting Shao, X 2020 (4)	0.79	[0.77; 0.81]
Omitting Cai, X-T 2021	0.79	[0.77; 0.81]
Omitting Olivera, A R 2017	0.79	[0.77; 0.81]
Omitting Liu, H 2024	0.79	[0.77; 0.81]
Omitting Jagannathan, R 2020 (1)	0.79	[0.78; 0.81]
Omitting Jagannathan, R 2020 (2)	0.79	[0.77; 0.81]
Omitting Jagannathan, R 2020 (3)	0.79	[0.78; 0.81]
Omitting Jagannathan, R 2020 (4)	0.79	[0.77; 0.81]
Omitting Savolainen, O 2017	0.79	[0.78; 0.81]
Omitting Sun, F 2009(1)	0.79	[0.77; 0.81]
Omitting Sun, F 2009(2)	0.79	[0.77; 0.81]
Omitting Lim, N K 2012 (1)	0.79	[0.78; 0.81]
Omitting Lim, N K 2012 (2)	0.79	[0.77; 0.81]
Omitting Lim, N K 2012 (3)	0.79	[0.77; 0.81]
Omitting Wang, H 2020	0.79	[0.77; 0.81]
Omitting Tan, C 2022	0.79	[0.77; 0.81]
Omitting Lindström, J 2003	0.79	[0.77; 0.81]
Omitting Woo, Y C 2016	0.79	[0.77; 0.81]
Omitting Hu, H 2020	0.79	[0.77; 0.81]
Omitting Sun, Q 2023	0.79	[0.77; 0.81]
Omitting Asgari, S 2021	0.79	[0.77; 0.81]
Omitting Yan Qun 2020	0.79	[0.77; 0.81]
Omitting Heianza, Y 2012 (1)	0.79	[0.77; 0.81]
Omitting Heianza, Y 2012 (2)	0.79	[0.77; 0.81]
Omitting Heianza, Y 2012 (3)	0.79	[0.77; 0.81]
Omitting Heianza, Y 2012 (4)	0.79	[0.77; 0.81]
Omitting Fu Xiaomin 2023	0.79	[0.77; 0.81]
Omitting Chen, L 2010	0.79	[0.77; 0.81]
Omitting Asgari, S 2021	0.79	[0.77; 0.81]
Omitting Zhu Xiaoyue 2023 (1)	0.79	[0.77; 0.81]
Omitting Zhu Xiaoyue 2023 (2)	0.79	[0.77; 0.81]
Omitting Liu, H 2024	0.79	[0.77; 0.81]
Omitting Zhu Xiaoyue 2023 (1)	0.79	[0.77; 0.81]
Omitting Zhu Xiaoyue 2023 (2)	0.79	[0.77; 0.81]
Omitting Liu, H 2024	0.79	[0.77; 0.81]
Omitting Zhu Xiaoyue 2023 (1)	0.79	[0.77; 0.81]
Omitting Zhu Xiaoyue 2023 (2)	0.79	[0.77; 0.81]
Omitting Zhu Xiaoyue 2023 (1)	0.79	[0.77; 0.81]
Omitting Zhu Xiaoyue 2023 (2)	0.79	[0.77; 0.81]
Omitting Olivera, A R 2017 (1)	0.79	[0.77; 0.81]
Total	0.79	[0.77; 0.81]

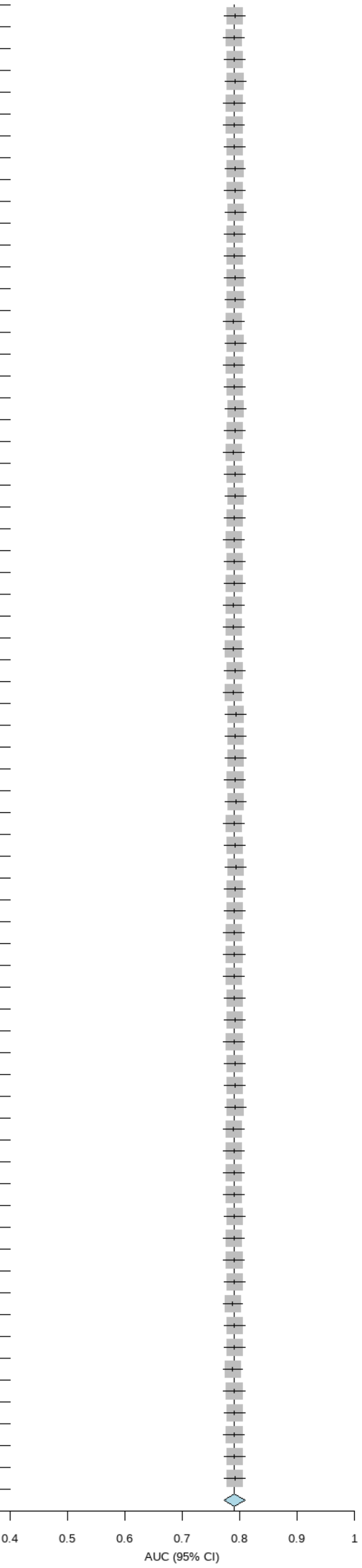

Figure S1

Sensitivity Analysis of Internal Validation

Omitted Study	AUC	95% CI
Omitting Han Xu 2017	0.79	[0.77; 0.81]
Omitting Sun Feng 2013	0.79	[0.77; 0.81]
Omitting Cai Xintian 2020	0.79	[0.77; 0.81]
Omitting Wen Jiangping 2016 (1)	0.79	[0.77; 0.81]
Omitting Wen Jiangping 2016 (2)	0.79	[0.77; 0.81]
Omitting Lei Qinghua 2023	0.79	[0.77; 0.81]
Omitting Su Ping 2017 (1)	0.79	[0.77; 0.81]
Omitting Su Ping 2017 (2)	0.79	[0.77; 0.81]
Omitting Yang Yang 2016	0.79	[0.77; 0.81]
Omitting Zhang Hongyan 2016 (1)	0.79	[0.77; 0.81]
Omitting Zhang Hongyan 2016 (2)	0.79	[0.77; 0.81]
Omitting Zhu Xiaoyue 2023 (1)	0.79	[0.77; 0.81]
Omitting Zhu Xiaoyue 2023 (2)	0.79	[0.77; 0.81]
Omitting Chen Yun 2018	0.79	[0.77; 0.81]
Omitting Ma Yujia 2020	0.79	[0.77; 0.81]
Omitting Liu Shaobo 2020	0.79	[0.77; 0.81]
Omitting Lee, H A 2024	0.79	[0.77; 0.81]
Omitting Li, Y 2019	0.79	[0.77; 0.81]
Omitting Hafezi, S G 2024	0.79	[0.77; 0.81]
Omitting Hu, H 2020	0.79	[0.77; 0.81]
Omitting Hu, H 2018 (1)	0.79	[0.77; 0.81]
Omitting Hu, H 2018 (2)	0.79	[0.77; 0.81]
Omitting Xu, J 2024 (1)	0.79	[0.78; 0.81]
Omitting Xu, J 2024 (2)	0.79	[0.77; 0.81]
Omitting Xu, J 2024 (3)	0.79	[0.77; 0.81]
Omitting Shao, X 2020 (1)	0.79	[0.77; 0.81]
Omitting Shao, X 2020 (2)	0.79	[0.77; 0.81]
Omitting Shao, X 2020 (3)	0.79	[0.77; 0.81]
Omitting Shao, X 2020 (4)	0.79	[0.77; 0.81]
Omitting Cai, X-T 2021	0.79	[0.77; 0.81]
Omitting Olivera, A R 2017	0.79	[0.77; 0.81]
Omitting Liu, H 2024	0.79	[0.77; 0.81]
Omitting Jagannathan, R 2020 (1)	0.79	[0.78; 0.81]
Omitting Jagannathan, R 2020 (2)	0.79	[0.77; 0.81]
Omitting Jagannathan, R 2020 (3)	0.79	[0.78; 0.81]
Omitting Jagannathan, R 2020 (4)	0.79	[0.77; 0.81]
Omitting Savolainen, O 2017	0.79	[0.78; 0.81]
Omitting Sun, F 2009(1)	0.79	[0.77; 0.81]
Omitting Sun, F 2009(2)	0.79	[0.77; 0.81]
Omitting Lim, N K 2012 (1)	0.79	[0.78; 0.81]
Omitting Lim, N K 2012 (2)	0.79	[0.77; 0.81]
Omitting Lim, N K 2012 (3)	0.79	[0.77; 0.81]
Omitting Wang, H 2020	0.79	[0.77; 0.81]
Omitting Tan, C 2022	0.79	[0.77; 0.81]
Omitting Lindström, J 2003	0.79	[0.77; 0.81]
Omitting Woo, Y C 2016	0.79	[0.77; 0.81]
Omitting Hu, H 2020	0.79	[0.77; 0.81]
Omitting Sun, Q 2023	0.79	[0.77; 0.81]
Omitting Asgari, S 2021	0.79	[0.77; 0.81]
Omitting Yan Qun 2020	0.79	[0.77; 0.81]
Omitting Heianza, Y 2012 (1)	0.79	[0.77; 0.81]
Omitting Heianza, Y 2012 (2)	0.79	[0.77; 0.81]
Omitting Heianza, Y 2012 (3)	0.79	[0.77; 0.81]
Omitting Heianza, Y 2012 (4)	0.79	[0.77; 0.81]
Omitting Fu Xiaomin 2023	0.79	[0.77; 0.81]
Omitting Chen, L 2010	0.79	[0.77; 0.81]
Omitting Asgari, S 2021	0.79	[0.77; 0.81]
Omitting Zhu Xiaoyue 2023 (1)	0.79	[0.77; 0.81]
Omitting Zhu Xiaoyue 2023 (2)	0.79	[0.77; 0.81]
Omitting Liu, H 2024	0.79	[0.77; 0.81]
Omitting Zhu Xiaoyue 2023 (1)	0.79	[0.77; 0.81]
Omitting Zhu Xiaoyue 2023 (2)	0.79	[0.77; 0.81]
Omitting Liu, H 2024	0.79	[0.77; 0.81]
Omitting Zhu Xiaoyue 2023 (1)	0.79	[0.77; 0.81]
Omitting Zhu Xiaoyue 2023 (2)	0.79	[0.77; 0.81]
Omitting Zhu Xiaoyue 2023 (1)	0.79	[0.77; 0.81]
Omitting Zhu Xiaoyue 2023 (2)	0.79	[0.77; 0.81]
Omitting Olivera, A R 2017 (1)	0.79	[0.77; 0.81]
Total	0.79	[0.77; 0.81]

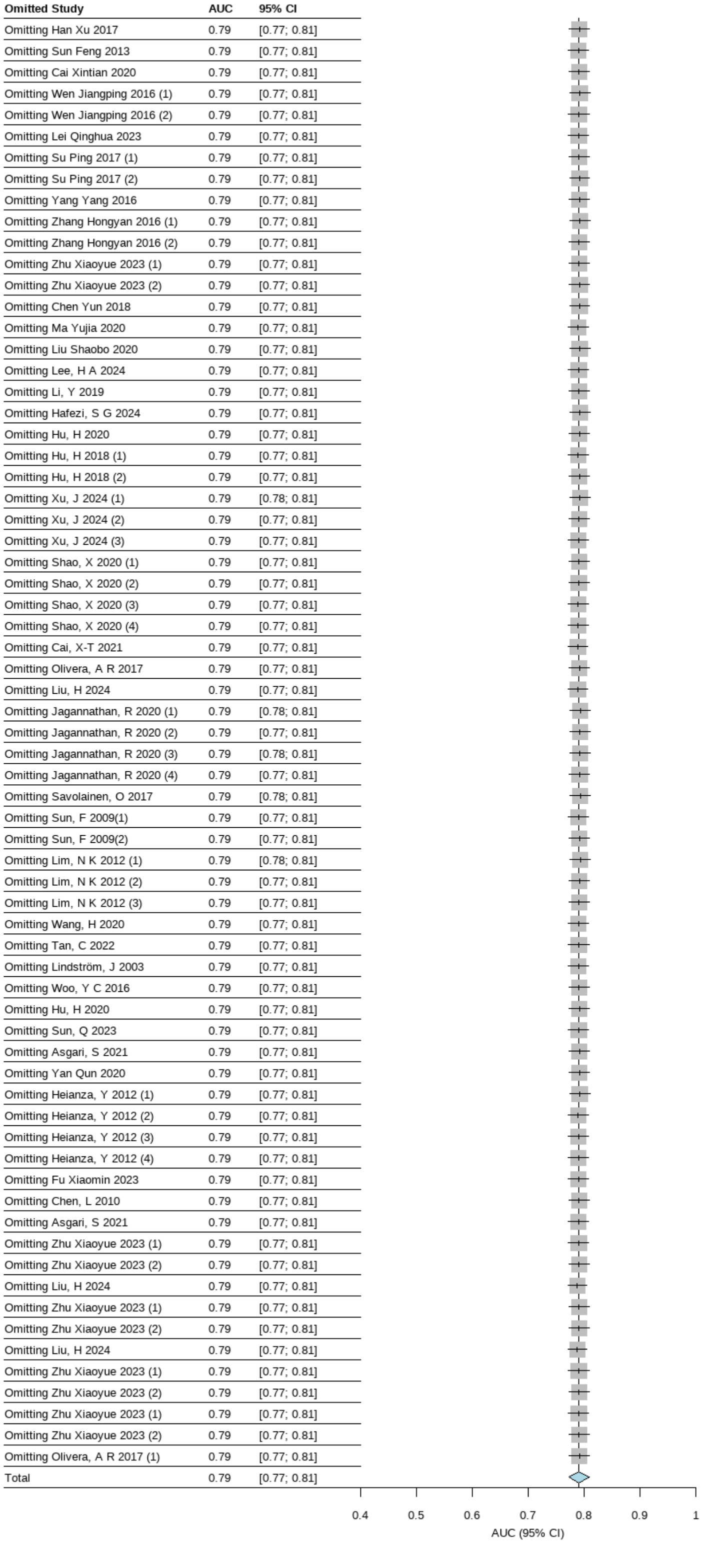

Figure S2

Sensitivity Analysis of External Validation

Omitted Study	AUC	95% CI
Omitting Sun Feng 2013	0.80	[0.77; 0.83]
Omitting Sun Feng 2013	0.80	[0.76; 0.83]
Omitting Sun Feng 2013	0.80	[0.77; 0.83]
Omitting Hippisley-Cox J 2009 (1)	0.80	[0.77; 0.83]
Omitting Hippisley-Cox J 2009 (2)	0.80	[0.76; 0.83]
Omitting Lee, H A 2024	0.80	[0.77; 0.83]
Omitting Lee, H A 2024	0.80	[0.77; 0.83]
Omitting Edlitz, Y 2022 (1)	0.81	[0.77; 0.84]
Omitting Edlitz, Y 2022 (2)	0.81	[0.78; 0.84]
Omitting Heianza, Y 2012 (1)	0.80	[0.77; 0.84]
Omitting Heianza, Y 2012 (2)	0.80	[0.76; 0.83]
Omitting Heianza, Y 2012 (3)	0.80	[0.76; 0.83]
Omitting Heianza, Y 2012 (4)	0.80	[0.76; 0.83]
Omitting Xu, J 2024 (1)	0.81	[0.77; 0.84]
Omitting Xu, J 2024 (2)	0.80	[0.77; 0.83]
Omitting Xu, J 2024 (3)	0.80	[0.76; 0.83]
Omitting Yan Qun 2020	0.81	[0.77; 0.84]
Omitting Asgari, S 2021	0.80	[0.77; 0.84]
Omitting Lindström, J 2003	0.80	[0.76; 0.83]
Omitting Chen, L 2010	0.81	[0.78; 0.84]
Omitting Chen, L 2010	0.80	[0.77; 0.84]
Total	0.80	[0.77; 0.83]

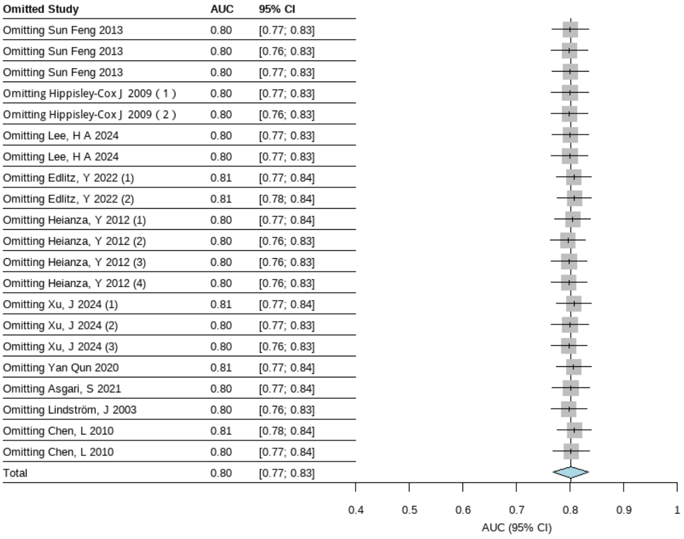

Figure S3
